# Multiway clustering with time-varying parameters

**DOI:** 10.1007/s00180-022-01294-5

**Published:** 2022-11-01

**Authors:** Roy Cerqueti, Raffaele Mattera, Germana Scepi

**Affiliations:** 1 Department of Social and Economic Sciences, Sapienza University of Rome, Rome, Italy; 2 School of Business, London South Bank University, London, UK; 3 GRANEM, University of Angers, Angers, France; 4 Department of Economics and Statistics, University of Naples “Federico II”, Naples, Italy

**Keywords:** Generalized Autoregressive Score, Dynamic Conditional Score, time-varying parameters, Time series clustering, Multiway data, Air quality

## Abstract

This paper proposes a clustering approach for multivariate time series with time-varying parameters in a multiway framework. Although clustering techniques based on time series distribution characteristics have been extensively studied, methods based on time-varying parameters have only recently been explored and are missing for multivariate time series. This paper fills the gap by proposing a multiway approach for distribution-based clustering of multivariate time series. To show the validity of the proposed clustering procedure, we provide both a simulation study and an application to real air quality time series data.

## Introduction

Clustering time series is an important tool for the analysis of real data in several contexts like biology, medicine, environmental sciences, engineering and finance. When clustering time series data, it is important to define a proper distance (Liao 2005). Distances based on the distributional characteristics of the time series are commonly considered (e.g. Nanopoulos et al. 2001; Wang et al. 2006; Fulcher and Jones 2014; D’Urso et al. 2017; Bastos and Caiado 2021). The idea of considering distribution characteristics is originally from Nanopoulos et al. (2001), that introduced the use of skewness and kurtosis in the clustering process. Later, Wang et al. (2006) and Fulcher and Jones (2014) proposed approaches of clustering based on multiple features, including the static first four moments. In particular, by using a partitioning clustering algorithm, D’Urso et al. (2017) proposed an approach based on time series’ extremes, using static parameters estimated from a Generalized Extreme Value (GEV) distribution. Similarly, Mattera et al. (2021) considered parameters estimated from a Skewed Generalized Error Distribution (SGED) to account for skewness and heavy tails. Recently, Bastos and Caiado (2021) considered a set of features for clustering financial time series where distribution characteristics were included. However, the use of distribution parameters is not limited to clustering time series data of economic and financial type. For example, Wang et al. (2011) proposed the use of parameters estimated from a Weibull distribution for clustering gene expression data.

The use of distribution parameters for clustering is well motivated by the high performances, in terms of clustering quality, that are witnessed by the previous studies.

According to previous studies (for an overview of time series clustering approaches see Maharaj et al. 2019), it is possible to classify time series with similar distribution parameters through a dissimilarity matrix computed on the difference of the estimated parameters in the clustering algorithm.

As highlighted by time series analysis studies, the use of static distribution parameters may not work with real time series data. The statistical models for time series with time-varying parameters have been categorized by Cox (1981) into two main classes, namely the observation-driven and the parameter-driven models. We focus our attention on the first type of models. In the observation-driven models, the time variation of the parameters is modeled through autoregressive approaches, where the parameters at a given time *t* are function of lagged values. This approach, that simplifies the likelihood evaluation, is very popular in the applied statistics and econometrics studies (e.g. see Creal et al. 2013; Harvey 2013; Harvey and Sucarrat 2014; Caivano and Harvey 2014; Koopman et al. 2016). Examples of observation-driven models are the ARCH (Engle 1982) and GARCH of Bollerslev (1986) for the variance, the Autoreressive Conditional Skewness (ARCS) of Harvey and Siddique (1999) for the skewness, the ARCSK of León et al. (2005) for modeling time variation in both skewness and kurtosis. More recently, Creal et al. (2013) proposed a very general approach to model time variation of the parameters for any kind of probability distribution. They developed a new statistical model, called Generalized Autoregressive Score (GAS), using the score function of the specified density as the source of time variation in the model’s parameters.

Despite clustering techniques, based on time series’ distribution characteristics, have been extensively studied, approaches based on time-varying parameters have only recently been explored in Cerqueti et al. (2021, 2022).

However, these two contributions have some weaknesses. The approach proposed in Cerqueti et al. (2021) is based on the selection of a target parameter. Although in some cases it can be of interesting to study clusters obtained according to a single distributional feature (e.g. the variance or the skewness), this approach can be less accurate when alternative features have their relevance in grouping the time series. Cerqueti et al. (2022) overcome the problem related to the selection of the target parameter by using more parameters jointly, focusing on the use of unconditional and conditional quantities in the clustering process. We have to acknowledge that the proposed unconditional distribution-based clustering provides results that are very close to the static parameters’ ones, even if the clustering interpretation is much more interesting. Most importantly, none of the two approaches can handle the case of multivariate time series.

In this paper, we propose a multiway clustering approach considering multiple time-varying parameters jointly in the definition of the clusters. We note that, with univariate time series with time-varying parameters the structure of the data is a 3D tensor, while with multivariate ones, it is a 4D tensor. According to the previous studies, we estimate the time-varying parameters with the GAS model.

To show the validity of the proposed multiway clustering procedure, we provide a simulation study with both univariate and multivariate time series. Moreover, we also show an application to real multivariate air pollution time series data. In particular, we aim identifying cities characterized by the same temporal evolution of air pollution, considering the Particular Matter (PM) time series variables as air quality indicators.

Studying air pollution clusters is important for policy makers. Indeed, there is a clear evidence that the presence of poor air quality leads to adverse effects on human health (e.g. see Dominici et al. 2003; Anderson et al. 2012). In particular, there is a strong association between PM and respiratory and cardiovascular diseases (see Rajagopalan et al. 2018). Moreover, there is a significant association between high levels of air pollution and the number of COVID-19 cases (Copat et al. 2020). Since the exposure to PM is dangerous to human health, policy makers of local governments take particularly into account monitoring of air quality (e.g. see Gao et al. 2011). In this framework, cluster analysis in an important tool for detecting groups of regions and/or cities with the same levels of air pollution (for a review see Govender and Sivakumar 2020).

Our analysis suggests the relevance of the proposed clustering approach in the development of public policies aimed at reducing the environmental impact in specific cities and/or geographical areas.

The paper is structured as follows. In Sect. 2, we describe the multiway clustering procedure in detail. In particular, in Sect. 2.1 we introduce preliminaries and notation and in Sect. 2.2 we show the proposed clustering procedure. Sections 2.3 and 2.4 discuss two particular cases with time-varying parameters estimated from a Gaussian and Generalized-t distributions. Section 3 provides experimental results with simulated data, while in Sect. 4 we show the empirical relevance of the proposed approach in the context of environmental quality monitoring. Final remarks with possible future research directions are discussed in the last section.

## Multiway clustering with time-varying parameters

Although many studies discussed the time-varying parameters’ evidence and there are a lot of statistical tools developed for modeling time variation in the parameters (e.g. see León et al. 2005; Harvey 2013; Creal et al. 2013; Harvey and Sucarrat 2014; Caivano and Harvey 2014), a clustering approach based on time-varying parameters has only recently been explored.

In what follows we propose a clustering approach for multivariate time series based on a multi steps algorithm (see e.g. Košmelj 1986; Košmelj and Batagelj 1990). We put our-self in the *Relationship Matrices Analysis* framework (for a clear illustration of such an approach, see e.g. D’Urso 2004), where the dissimilarity between units is determined by considering a relationship matrix (e.g. correlation, distance, etc.) between pairs of elements.

### Preliminaries and notation

Let *N* be the number of statistical units and *K* the number of time series variables of length *T*. The distribution-based clustering approaches have mainly been developed for clustering univariate time-series, i.e. in presence of *N* statistical units and $K=1$ variable. By denoting the single $K=1$ variable as $y_t$, we have that $y_{n,t}$ represents the values of the time series variable $y_t$ for the *n*-th statistical unit.

To assist the reader, we firstly present the notation used with univariate time series characterized by static distribution parameters. Let ${\mathbf {Y}} =\{y_{n,t}: n=1,\ldots ,N; \;t=1,\ldots ,T\}$ be the dataset matrix containing the *N* univariate time series—i.e., the statistical units—whose *n*-th element is $\{y_{n,t}: t=1,\ldots ,T\}$. Therefore:

1 $$\begin{aligned} {\mathbf {Y}} = \begin{bmatrix} y_{1,1} &\quad \dots &\quad y_{n,1} &\quad \dots &\quad y_{N,1}\\ \vdots &\quad \dots &\quad \vdots &\quad \dots &\quad \vdots \\ y_{1,t} &\quad\dots &\quad y_{n,t} &\quad \dots &\quad y_{N,t} \\ \vdots &\quad \dots &\quad\vdots &\quad \vdots &\quad \vdots \\ y_{1,T} &\quad \dots &\quad y_{n,T} &\quad \dots &\quad y_{N,T} \end{bmatrix} \end{aligned}$$

Let us suppose that each column of the (1) is generated by a probability density function $p(\cdot )$ characterized by the presence of *J* parameters, so that we call $f_{n,j}$ the *j*-th static distribution parameter associated to the *n*-th statistical units. For example, in the case $p(\cdot )$ follows a Gaussian distribution, we have $J=2$ parameters, so that $f_{n,1}=\mu _n$ and $f_{n,2}=\sigma ^2_n$ are, respectively, the mean and the variance of the *n*-th statistical unit. Therefore, the number of *J* parameters depends on the underlying distributional assumption. In presence of a general $p(\cdot )$ density, a distribution-based clustering considers the following $(N \times J)$ matrix $\mathbf {F}$ as the input of the algorithm:

2 $$\begin{aligned} \mathbf {F} = \begin{bmatrix} f_{1,1} &\quad\dots &\quad f_{1,j} &\quad \dots &\quad f_{1,J} \\ \vdots &\quad \dots &\quad\vdots &\quad \vdots &\quad \vdots \\ f_{n,1} &\quad\dots &\quad f_{n,j} &\quad \dots &\quad f_{n,J} \\ \vdots &\quad \dots &\quad\vdots &\quad \vdots &\quad \vdots \\ f_{N,1} &\quad \dots &\quad f_{N,j} &\quad \dots &\quad f_{N,J} \\ \end{bmatrix} \end{aligned}$$

where the distribution parameters $f_{n,j}$ can be estimated with maximum likelihood.

In the case of $K\ge 2$ multivariate time series, we define $y_{n,k,t}$ $(n=1,\dots ,N; k=1\,\dots ,\;K, t=1,\dots ;T)$ the value of the *k*-th variable at time *t* for the *n*-th statistical unit. Therefore, in the case of multivariate time series, the matrix (1) becomes a 3D tensor:

3 $$\begin{aligned} \tilde{{\mathbf {Y}}} = \left\{ y_{n,k,t}: n=1, \dots , N; \,k=1,\dots , \, K; t=1,\dots , T\right\} \end{aligned}$$

By considering static distribution parameters with $K\ge 2$, we have that the matrix (2) has a 3D tensorial representation with the elements $f_{n,k,j}$ representing the *j*-th static distribution parameter associated to the *k*-th variable of the *n*-th unit.

We are now in the position to introduce our contribution to the methodological setting of the time-varying parameters in the mumtivariate time-series context. Specifically, we introduce time variation in the parameters of multivariate time series. In this case the $f_{n,k,j}$s in the 3D tensorial representation are time series themselves. Therefore, by considering time-varying parameters for multivariate time series (3), we have that the matrix (2) is the following 4D tensor called $\tilde{\mathbf {F}}$:

4 $$\begin{aligned} \tilde{\mathbf {F}} = \left\{ f_{n,k,j,t}: n=1, \dots , N; k=1,\dots ,K; j=1, \dots ,J; t=1,\dots , T\right\} \end{aligned}$$

where $f_{n,k,j,t}$ denotes the *j*-th distribution parameter for the *k*-th variable of the *n*-th statistical unit at time *t*. Clearly, the general formulation in (4) includes also the univariate time-dependent case ($K=1$) and the static univariate case ($K=1$ and $T=1$).

In this paper, starting from the multivariate time series data (3), we first estimate the terms appearing in equation (4). Then, we consider the multivariate time-varying parameters as the input of the clustering procedure. In order to model and estimate the time-varying parameters in (4), following previous studies, we use the Generalized Autoregressive Score (GAS) model of Creal et al. (2013). For details about the GAS model see the “Appendix 2”. Therefore, the estimated time-varying parameters $\hat{f}_{n,k,j,t}$ are used as the input of the clustering procedure. The similarity between statistical units is defined by the degree to which the distribution parameters, for each variable, vary over time.

### The clustering procedure

The proposed clustering procedure, inspired from the double-step approaches for clustering longitudinal data (Košmelj 1986; Košmelj and Batagelj 1990), can be outlined as follows.

Let $f_{n,k,j,t}$ be the realization of the *j*-th time-varying parameter associated to the *k*-th variable for the *n*-th statistical unit at time *t* (4); we define $\rho _{n,k,j,l}$ as the estimated auto-correlation at lag $l (l=1,\dots ,L)$ of the *j*-th time-varying parameter associated to the *k*-th variable of the *n*-th unit.

In the first step of the clustering procedure we compute $N \times K$ distance matrices ${\mathbf {D}}_{n,k} = \left\{ d_{n, k, j, j^{\prime }}: j, j^{\prime }=1,\dots , J ; j \ne j^{\prime }\right\}$, for each $n=1,\dots ,N; \;k=1,\dots ,K$. In line with previous studies (see e.g. Cerqueti et al. 2021), we consider an ACF-based distance between two pairs of time-varying parameters *j* and $j\prime$:

5 $$\begin{aligned} d_{n,k,j,j\prime } = \sqrt{\sum _{\ell =1}^{L} \left( \rho _{n,k,j,l}- \rho _{n,k,j\prime ,l}\right) ^2} \end{aligned}$$

Therefore, each matrix ${\mathbf {D}}_{n,k}$ can be written as follows:

6 $$\begin{aligned} {{\mathbf {D}}_{n,k}} = \begin{bmatrix} 0 &{} d_{n,k,1,2} &{} \dots &{} d_{n,k,1,J} \\ d_{n,k,2,1} &{} 0 &{} \dots &{} d_{n,k,2,J} \\ \vdots &{} \vdots &{} \ddots &{} \vdots \\ d_{n,k,J,1} &{} d_{n,k,J,2} &{} \dots &{} 0\\ \end{bmatrix} \end{aligned}$$

Note that each ${\mathbf {D}}_{n,k}$ is a squared matrix of order *J* and it is symmetric with a null diagonal. In the second step of the procedure we aim to cluster the *N* statistical units on the basis of a dissimilarity measure among the matrices ${\mathbf {D}}_{n,k}$. Let $\mathbf {L}_{n,k}$ be the lower triangular of ${\mathbf {D}}_{n,k}$:

7 $$\begin{aligned} {\mathbf {L}_{n,k}} = \begin{bmatrix} 0 &{} 0 &{} \dots &{} 0 \\ d_{n,k,2,1} &{} 0 &{} \dots &{} 0\\ \vdots &{} \vdots &{} \ddots &{} \vdots \\ d_{n,k,J,1} &{} d_{n,k,J,2} &{} \dots &{} 0\\ \end{bmatrix} \end{aligned}$$

Since each ${\mathbf {D}}_{n,k}$ is squared and symmetric with a null diagonal, we can vectorize its lower triangular $\mathbf {L}_{n,k}$ without losing information. The vectorized lower triangular, called ${\text{vec}}(\mathbf {L}_{n,k})$, can be written as follows:

8 $$\begin{aligned} {{\text{vec}}\left( \mathbf {L}_{n,k}\right) }= \begin{bmatrix} d_{n,k,2,1} &{} \dots &{} &{} d_{n,k,J,J-1}\\ \end{bmatrix} \end{aligned}$$

Note that ${\text{vec}}\left( \mathbf {L}_{n,k}\right)$ has a length equal to $[J(J-1)]/2$.

In the second step, we define, for each *k*-th variable, the matrix $\mathbf {X}_k$ whose rows are given by the *N* vectors ${\text{vec}}(\mathbf {L}_{n,k})$:

9 $$\begin{aligned} {\mathbf {X}_{k}} = \begin{bmatrix} d_{1,k,2,1} &{} \dots &{} d_{1,k,J,J-1} \\ \vdots &{} \vdots &{} \vdots \\ d_{n,k,2,1} &{} \dots &{} d_{n,k,J,J-1} \\ \vdots &{} \vdots &{}\vdots \\ d_{N,k,2,1} &{} \dots &{} d_{N,k,J,J-1} \\ \end{bmatrix} \end{aligned}$$

Therefore, each $\mathbf {X}_{k}$ is of dimension $N \times [J(J-1)]/2$. The generic element of $\mathbf {X}_{n}$ is denoted by $x_{k,n,r}$ $(r=1,\dots ,[J(J-1)]/2)$. Then, we can define the *k*-th ${\mathbf {D}}_{k}$ distance matrix with dimension $N \times N$, whose generic element $d_{k, n, n\prime }$ can be written as follows:

10 $$\begin{aligned} d_{k, n, n\prime } = \sqrt{\sum _{r=1}^{[J(J-1)]/2} \left( x_{k,n,r} - x_{k,n\prime , r}\right) ^2} \end{aligned}$$

Each *k*-th distance ${\mathbf {D}}_k$ contains the information about dissimilarity of the *N* statistical units computed considering the *k*-th variable.

In order to consider the information included in each of the *K* variables jointly, in the third phase we compute a synthesis of the *K* distance matrices ${\mathbf {D}}_k$ through the DISTATIS algorithm (for details see “Appendix 3”). The resulting consensus squared Euclidean distance matrix $\varvec{\tilde{D}}$ (37) has as generic element $\tilde{d}_{n,n^\prime }$ and represents the synthesis of the *K* distances in (10).

In the last step, we use the resulting consensus distance matrix in the Partition Around Medoid (PAM) (Kaufman and Rousseeuw 1990) algorithm to obtain the clusters. The PAM algorithm is based on the minimization of the squared elements of matrix $\varvec{\tilde{D}}$, being one of the unit the centroid. In formulas, we have the following minimization problem:

11 $$\begin{aligned} \min : \sum _{n=1}^{N} \sum _{c=1}^{C} \tilde{d}^2_{n,c}. \end{aligned}$$

Clearly, the univariate time series clustering is a special case where $K=1$. In a this particular framework, we deal where a 3D tensor with the three dimensions are represented by *N* statistical units, *J* parameters and *T* time. Essentially, the clustering procedure in the univariate framework is very similar to the one explained so far, the difference is that we do not need to compute a consensus matrix.
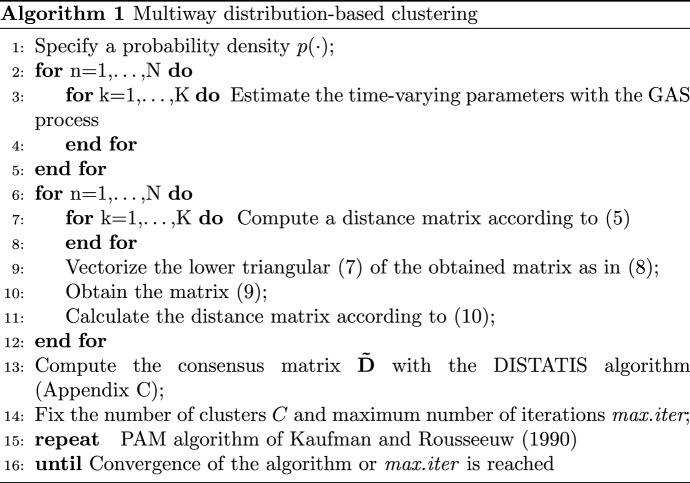


### Example with Gaussian density

Let us consider the data structure shown in (3) where each $y_{n,k,t}$ time series follows a Gaussian distribution with time-varying parameters. In this case, the predictive density can be written as follows:

12 $$\begin{aligned} p(y_{n,k,t} \vert \mu _{n,k,t}, \sigma ^2_{n,k,t}, {\mathcal {F}}_{n,k,t}; \theta _{n,k}) = \frac{1}{{\sigma _{n,k,t} \sqrt{2\pi } }}e^{{{ - \left( {y_{n,k,t} - \mu _{n,k,t} } \right) ^2 } / {2\sigma ^2_{n,k,t} }}} \end{aligned}$$

where $\mu _{n,k,t}$ is the time-varying mean, $\sigma ^2_{n,k,t}$ the time-varying variance, ${\mathcal {F}}_{n,k,t}$ is the information set and $\theta _{n,k} = \left[ \omega _{n,k}, {\text {diag}}\left( {\mathbf {A}}_{n,k}\right) , {\text {diag}}\left( {{\mathbf {B}}}_{n,k}\right) \right]$ contains the parameters estimated by the following Gaussian-GAS(1,1) process:

$$\begin{aligned} f_{n,k,t} = \omega _{n,k} + {\mathbf {A}}_{n,k} s_{n,k,t-1} + {\mathbf {B}}_{n,k} f_{n,k,t-1} \end{aligned}$$

given $f_{n,k,t}$ the vector containing time-varying parameters $f_{n,k,j,t} = \left[ f_{n,k,1,t}, f_{n,k,2,t}\right] = \left[ \mu _{n,k,t}, \sigma ^2_{n,k,t}\right]$ and $s_{n,k,t}$ the scaled score with conditional scores equal to:

13 $$\begin{aligned} \nabla _{n,k,1,t} = \frac{(y_{n,k,t}-\mu _{n,k,t})}{\sigma ^2_{n,k,t}} \end{aligned}$$

14 $$\begin{aligned} \nabla _{n,k,2,t} = \frac{(y_{n,k,t}-\mu _{n,k,t})^2}{2\sigma ^4_{n,k,t}} - \frac{T}{2\sigma ^2_{n,k,t}} \end{aligned}$$

where $\nabla _{n,k,1,t}$ is the score related to the time-varying mean (i.e. $j=1$) and $\nabla _{n,k,2,t}$ is the score related to the time-varying variance (i.e. $j=2$). In summary, the model’s variables and parameters are:

$$\begin{aligned} f_{n,k,t} = \begin{pmatrix} \mu _{n,k,t} \\ \sigma ^2_{n,k,t} \end{pmatrix}, \quad \omega = \begin{pmatrix} \omega _{n,k,1} \\ \omega _{n,k,2} \end{pmatrix} \text {,}\\ {\mathbf {A}} = \begin{pmatrix} a_{n,k,1} &{} 0 \\ 0 &{} a_{n,k,2} \end{pmatrix} \quad \text {and} \quad {\mathbf {B}} = \begin{pmatrix} b_{n,k,1} &{} 0\\ 0 &{} b_{n,k,2} \end{pmatrix} \end{aligned}$$

In the univariate case (i.e. $K=1$), we compute the matrices ${\mathbf {D}}_n$^1^ according to formula (5). The matrices ${\mathbf {D}}_n$ in the case of Gaussian distribution can be written as follows:

$$\begin{aligned} {\mathbf {D}}_n = \begin{bmatrix} d_{\mu _n, \mu _n} &{} d_{\mu _n, \sigma ^2_n} \\ d_{\mu _n, \sigma ^2_n} &{} d_{\sigma ^2_n, \sigma ^2_n} \\ \end{bmatrix} = \begin{bmatrix} 0 &{} d_{\mu _n, \sigma ^2_n} \\ d_{\mu _n, \sigma ^2_n} &{} 0 \\ \end{bmatrix} \end{aligned}$$

The value $d_{\mu _n, \sigma ^2_n}$ summarises the difference among the $J=2$ parameters. Two units *n* and $n\prime$ can be considered similar if $d_{\mu _n, \sigma ^2_n}$ is close to $d_{\mu _{n\prime }, \sigma ^2_{n\prime }}$. According to the procedure highlighted so far, we vectorize the lower triangular of each ${\mathbf {D}}_n$. In the peculiar case of Gaussian density, however, the vectorization results into a single point, i.e. $d_{\mu _n, \sigma ^2_n}$. Therefore, we concatenate the values of ${\text{vec}}\left( \mathbf {L}_n\right)$ as follows:

15 $$\begin{aligned} \mathbf {X} = \begin{bmatrix} d_{\mu _1, \sigma ^2_1} \\ d_{\mu _2, \sigma ^2_2} \\ \vdots \\ d_{\mu _n, \sigma ^2_n} \\ \vdots \\ d_{\mu _N, \sigma ^2_N} \end{bmatrix} \end{aligned}$$

obtaining a vector of dimension $N\times 1$. An Euclidean distance among the values of the vector $\mathbf {X}$ is the distance matrix used for the implementation of the PAM algorithm. Note that these arguments apply when any probability distribution with $J=2$ parameters is specified^2^.

Let us now analyse the case in which *K* multivariate time series are studied with their time-varying parameters jointly. For each *N* units, we consider the generic *k*-th distance matrix:

$$\begin{aligned} {\mathbf {D}}_{n,k} =\begin{bmatrix} 0 &{} d_{\mu _{n,k}, \sigma ^2_{n,k}} \\ d_{\mu _{n,k}, \sigma ^2_{n,k}} &{} 0 \\ \end{bmatrix}. \end{aligned}$$

Then, we vectorize the lower triangular of each *k*-th matrix. By concatenating these values we obtain the following vector:

16 $$\begin{aligned} \mathbf {X}_k = \begin{bmatrix} d_{\mu _{1,k}, \sigma ^2_{1,k}} \\ d_{\mu _{2,k}, \sigma ^2_{2,k}} \\ \vdots \\ d_{\mu _{n,k}, \sigma ^2_{n,k}} \\ \vdots \\ d_{\mu _{N,k}, \sigma ^2_{N,k}} \end{bmatrix} \end{aligned}$$

Each $\mathbf {X}_k$ is used to define a dissimilarity matrix ${\mathbf {D}}_k$. To obtain a synthesis, we apply the DISTATIS algorithm of Abdi et al. (2005). Hence, we find a consensus matrix $\varvec{\tilde{D}}$ that is then employed as the distance in the PAM algorithm (11).

### Example with Generalized-t density

Let us consider the data structure shown in (3) where each $y_{n,k,t}$ time series follows a Generalized-t distribution with $J=3$ time-varying parameters. The density of a Generalized-t distribution with time-varying parameters can be written as follows:

17 $$\begin{aligned}&p(y_{n,k,t} \mid \mu _{n,k,t}, \phi _{n,k,t}, \nu _{n,k,t}, {\mathcal {F}}_{n,k,t}; \theta _{n,k}) \nonumber \\&=\frac{\Gamma \left( \frac{\nu _{n,k,t} + 1}{2}\right) }{\Gamma \left( \frac{\nu _{n,k,t}}{2}\right) \phi _{n,k,t} \sqrt{\pi \nu _{n,k,t}}} \left( 1 + \frac{(y_{n,k,t} - \mu _t)^2}{\nu _{n,k,t} \phi _{n,k,t}}\right) ^{\frac{\nu _{n,k,t}+1}{2}} \end{aligned}$$

with location $\mu _{n,k,t}$, scale $\phi _{n,k,t}$ and shape $\nu _{n,k,t}>2$, ${\mathcal {F}}_{n,k,t}$ is the information set and $\theta _{n,k} = \left[ \omega _{n,k}, {\text {diag}}\left( {\mathbf {A}}_{n,k}\right) , {\text {diag}}\left( {\mathbf {B}}_{n,k}\right) \right]$ contains the parameters estimated by the following t-GAS(1,1) process:

$$\begin{aligned} f_{n,k,t} = \omega _{n,k} + {\mathbf {A}}_{n,k} s_{n,k,t-1} + {\mathbf {B}}_{n,k} f_{n,k,t-1} \end{aligned}$$

where differently from the Gaussian example, $f_{n,k,j,t} = \left[ f_{n,k,1,t}, f_{n,k,2,t}, f_{n,k,3,t}\right] = \left[ \mu _{n,k,t}, \phi _{n,k,t}, \nu _{n,k,t}\right]$. The scaled scores, $s_{n,k,t}$, are equal to:

18 $$\begin{aligned} \nabla _{n,k,1,t}= & {} \frac{\left( \nu _{n,k,t}+1\right) \left( y_{n,k,t}-\mu _{n,k,t}\right) }{\nu _{n,k,t} \phi _{n,k,t}\left( 1+\frac{\left( y_{n,k,t}-\mu _{n,k,t}\right) ^{2}}{\nu _{n,k,t} \phi _{n,k,t}}\right) } \end{aligned}$$

19 $$\begin{aligned} \nabla _{n,k,2,t}= & {} \frac{\left( \nu _{n,k,t}+1\right) \left( y_{n,k,t}-\mu _{n,k,t}\right) ^{2}}{2 \nu _{n,k,t} \phi _{n,k,t}^{2}\left( 1+\frac{\left( y_{n,k,t}-\mu _{n,k,t}\right) ^{2}}{\nu _{n,k,t} \phi _{n,k,t}}\right) }-\frac{1}{\phi _{n,k,t}} \end{aligned}$$

20 $$\begin{aligned} \nabla _{n,k,3,t}= & {} \frac{1}{2} \psi \left( \frac{\nu _{n,k,t}+1}{2}\right) -\frac{1}{2} \psi \left( \frac{\nu _{n,k,t}}{2}\right) -\frac{1}{2 \nu _{n,k,t}} \nonumber \\&-\frac{1}{2} \log \left( 1+\frac{\left( y_{n,k,t}-\mu _{n,k,t}\right) ^{2}}{\nu _{n,k,t} \phi _{n,k,t}}\right) +\frac{\left( \nu _{n,k,t}+1\right) \left( y_{n,k,t}-\mu _{n,k,t}\right) ^{2}}{2 \nu _{n,k,t}^{2} \phi _{n,k,t}\left( 1+\frac{\left( y_{n,k,t}-\mu _{n,k,t}\right) ^{2}}{\nu _{n,k,t} \phi _{n,k,t}}\right) } \end{aligned}$$

with $\psi (\cdot )$ being the Digamma function. Hence, $\nabla _{n,k,1,t}$ is the score related to the time-varying location (i.e. $j=1$), $\nabla _{n,k,2,t}$ is the score related to the time-varying scale (i.e. $j=2$) and $\nabla _{n,k,3,t}$ is the score related to the time-varying shape (i.e. $j=3$). Finally, the model’s variables and parameters are:

$$\begin{aligned}& f_{n,k,t} = \begin{pmatrix} \mu _{n,k,t} \\ \phi _{n,k,t} \\ \nu _{n,k,t} \end{pmatrix}, \quad \omega = \begin{pmatrix} \omega _{n,k,1} \\ \omega _{n,k,2} \\ \omega _{n,k,3} \end{pmatrix} ,\\ &{\mathbf {A}} = \begin{pmatrix} a_{n,k,1} &{} 0 &{} 0 \\ 0 &{} a_{n,k,2} &{} 0 \\ 0 &{} 0 &{} a_{n,k,3} \end{pmatrix} \quad \text {and} \quad {\mathbf {B}} = \begin{pmatrix} b_{n,k,1} &{} 0 &{} 0\\ 0 &{} b_{n,k,2} &{}0 \\ 0 &{} 0 &{} b_{n,k,3} \end{pmatrix} \end{aligned}$$

Let us discuss, first, the univariate case. We estimate the time-varying parameters by means of the t-GAS(1,1) process (29). Then, we compute the matrices ${\mathbf {D}}_n$ according to formula (5). The matrices ${\mathbf {D}}_n$ in the case of Generalized-t distribution can be written as follows:

$$\begin{aligned} {\mathbf {D}}_n = \begin{bmatrix} d_{\mu _n, \mu _n} &{} d_{\mu _n, \phi _n} &{} d_{\mu _n, \nu _n} \\ d_{\mu _n, \phi _n} &{} d_{\phi _n, \phi _n} &{} d_{\phi _n, \nu _n} \\ d_{\mu _n, \nu _n}&{} d_{\phi _n, \nu _n} &{} d_{\nu _n, \nu _n} \\ \end{bmatrix} = \begin{bmatrix} 0 &{} d_{\mu _n, \phi _n} &{} d_{\mu _n, \nu _n} \\ d_{\mu _n, \phi _n} &{} 0 &{} d_{\phi _n, \nu _n} \\ d_{\mu _n, \nu _n}&{} d_{\phi _n, \nu _n} &{} 0 \\ \end{bmatrix} \end{aligned}$$

According to the procedure highlighted so far, we vectorize the lower triangular of each ${\mathbf {D}}_n$. The vectorization results into the following vector:

21 $$\begin{aligned} {\text{vec}}\left( \mathbf {L}_n\right) = \begin{bmatrix} d_{\mu _n, \phi _n} \\ d_{\mu _n, \nu _n} \\ d_{\phi _n, \nu _n} \\ \end{bmatrix} \end{aligned}$$

Then, by concatenating the vectors ${\text{vec}}\left( \mathbf {L}_n\right)$ we have:

22 $$\begin{aligned} \mathbf {X} = \begin{bmatrix} d_{\mu _1, \phi _1} &{} \dots &{} d_{\mu _n, \phi _n} &{} \dots &{} d_{\mu _N, \phi _N} \\ d_{\mu _1, \nu _1} &{}\dots &{} d_{\mu _n, \nu _n} &{} \dots &{} d_{\mu _N, \nu _N}\\ d_{\phi _1, \nu _1} &{} \dots &{} d_{\phi _n, \nu _n} &{} \dots &{} d_{\phi _N, \nu _N}\\ \end{bmatrix} \end{aligned}$$

where each column of $\mathbf {X}$ represents the *n*-th statistical unit to be clustered and the rows are the dissimilarities among the time-varying parameters. An Euclidean distance among the columns of the matrix $\mathbf {X}$ is the distance matrix among the *N* units. Note that when the probability distribution has $J>2$ time-varying parameters, the vector $\mathbf {X}$ (15) becomes a matrix.

Let analyse the case in which *K* multivariate time series are jointly studied with their time-varying parameters. For each *n*-th unit, let us consider the *k*-th ACF-based distance matrices:

$$\begin{aligned} {\mathbf {D}}_{n,k} =\begin{bmatrix} 0 &{} d_{\mu _{n,k}, \phi _{n,k}} &{} d_{\mu _{n,k}, \phi _{n,k}}\\ d_{\mu _{n,k}, \phi _{n,k}} &{} 0 &{} d_{\mu _{n,k}, \nu _{n,k}} \\ d_{\mu _{n,k}, \phi _{n,k}} &{} d_{\mu _{n,k}, \phi _{n,k}} &{} 0 \\ \end{bmatrix} \end{aligned}$$

For each *k*-th variable, we vectorize the lower triangular. By concatenating these values we define the following matrix:

23 $$\begin{aligned} \mathbf {X}_k = \begin{bmatrix} d_{\mu _{1,k}, \phi _{1,k}} &{} \dots &{} d_{\mu _{n,k}, \phi _{n,k}} &{} \dots &{} d_{\mu _{N,k}, \phi _{N,k}} \\ d_{\mu _{1,k}, \nu _{1,k}} &{}\dots &{} d_{\mu _{n,k}, \nu _{n,k}} &{} \dots &{} d_{\mu _{N,k}, \nu _{N,k}}\\ d_{\phi _{1,k}, \nu _{1,k}} &{} \dots &{} d_{\phi _{n,k}, \nu _{n,k}} &{} \dots &{} d_{\phi _{N,k}, \nu _{N,k}}\\ \end{bmatrix} \end{aligned}$$

As in the example with Gaussian density, each $\mathbf {X}_k$ is used to define a dissimilarity matrix ${\mathbf {D}}_k$, whose general element is defined in (10). To obtain a synthesis of the *K* dissimilarity matrices, we apply the DISTATIS algorithm (see “Appendix 3”). Hence, we find a consensus matrix $\varvec{\tilde{D}}$ that is then employed as the distance in the PAM algorithm (11).

## Experimental results with simulated data

To show the validity of the proposed clustering procedure, we provide an application to simulated data. We generate several alternative simulation schemes. The simulation schemes are based on time series simulated from the following Gaussian-GAS processess:

24 $$\begin{aligned}&\omega _1=(0.0490,0.0154); \quad {\mathbf {A}}_1 = \begin{pmatrix} 0.0001 &{} 0\\ 0 &{} 0.0534 \end{pmatrix}; \quad {\mathbf {B}}_1 = \begin{pmatrix} 0.0485 &{} 0\\ 0 &{} 0.9891 \end{pmatrix} \end{aligned}$$

25 $$\begin{aligned}&\quad \omega _2=(0.0840,0.0456); \quad {\mathbf {A}}_2 = \begin{pmatrix} 0.00001 &{} 0\\ 0 &{} 0.0139 \end{pmatrix}; \quad {\mathbf {B}}_2 = \begin{pmatrix} 0.0660 &{} 0\\ 0 &{} 0.0968 \end{pmatrix} \end{aligned}$$

26 $$\begin{aligned}&\quad \omega _3=(0.0759,0.0095); \quad {\mathbf {A}}_3 = \begin{pmatrix} 0.0001 &{} 0\\ 0 &{} 0.0471 \end{pmatrix}; \quad {\mathbf {B}}_3 = \begin{pmatrix} 0.001 &{} 0\\ 0 &{} 0.9920 \end{pmatrix} \end{aligned}$$

27 $$\begin{aligned}&\quad \omega _4=(0.0686,0.0230); \quad {\mathbf {A}}_4 = \begin{pmatrix} 0.0001 &{} 0\\ 0 &{} 0.0755 \end{pmatrix}; \quad {\mathbf {B}}_4 = \begin{pmatrix} 0.0018 &{} 0\\ 0 &{} 0.9791 \end{pmatrix} \end{aligned}$$

with parameters calibrated on the basis of real time series data. In the case of univariate time series, i.e. with $K=1$, we provide 90 alternative simulation schemes, comparing the clustering accuracy assuming the following DGPs:

- DGPs I: *N*/2 time series of length *T* from (24) and *N*/2 time series of length *T* from (25);
- DGPs II: *N*/2 time series of length *T* from (24) and *N*/2 time series of length *T* from (26);
- DGPs III: *N*/2 time series of length *T* from (24) and *N*/2 time series of length *T* from (27);
- DGPs IV: *N*/2 time series of length *T* from (25) and *N*/2 time series of length *T* from (26);
- DGPs V: *N*/2 time series of length *T* from (25) and *N*/2 time series of length *T* from (27);
- DGPs VI: *N*/2 time series of length *T* from (26) and *N*/2 time series of length *T* from (27);

under three different sample sizes $N=\{10, 30, 60\}$ and with four time series’ lengths, namely $T=\{150,250, 500, 1000, 2000\}$. Therefore, we also evaluate how the performance of the clustering algorithm is affected by the number of statistical units *N* and the time series’ length *T*, considering six combinations of the alternative DGPs. For all the simulations we assume $C=2$ clusters.

The proposed clustering approach is compared with two clustering algorithms. The first benchmark is represented by a standard PAM approach, where cluster analysis is conducted considering the original time series rather than their time-varying parameters. Then, a second benchmark is represented by Cerqueti et al. (2021), that considers the auto-correlation of a target time-varying parameter for clustering. In the case of Gaussian density, we consider the Cerqueti et al. (2021) algorithm with both mean and variance targeting. Differently, the approach proposed in this paper jointly considers all the time-varying parameters in the clustering process.

The performances of the algorithms are compared in terms of adjusted Rand Index (ARI, Hubert and Arabie 1985), averaged over 100 trials as in Park and Jun (2009).

The results in the case of $N=10$ time series are shown in Table 1.

**Table 1:** Clustering results: average Adjusted Rand Index $(N=10; \; K=1)$

Clustering approach	Proposal	Target $\mu _t$	Target $\sigma ^2_t$	Benchmark
DGPs scenario I: models (24) and (25)				
$T=150$	0.0660	− 0.0103	0.0590	− 0.0261
$T=250$	0.1366	− 0.0175	0.1167	− 0.0120
$T=500$	0.3166	0.0018	0.2475	− 0.0119
$T=1000$	0.6561	− 0.0036	0.5872	0.0047
$T=2000$	0.8731	− 0.0021	0.8588	0.0053
DGPs scenario II: models (24) and (26)				
$T=150$	− 0.00347	− 0.0137	− 0.0113	0.0002
$T=250$	− 0.0105	0.0981	− 0.0103	0.02046
$T=500$	0.1615	0.3771	0.0921	0.2342
$T=1000$	0.6075	− 0.0195	0.1934	− 0.0754
$T=2000$	0.9854	0.4268	0.9622	− 0.0570
DGPs scenario III: models (24) and (27)				
$T=150$	0.0124	− 0.0080	0.0069	0.0052
$T=250$	0.0189	− 0.0070	0.0126	− 0.0087
$T=500$	0.0579	0.0059	0.0706	− 0.0057
$T=1000$	0.2542	0.0108	0.2141	− 0.0197
$T=2000$	0.4360	0.0793	0.4083	0.0017
DGPs scenario IV: models (25) and (26)				
$T=150$	0.0515	0.0289	0.0413	− 0.0046
$T=250$	0.1788	0.0111	0.1502	0.0028
$T=500$	0.4191	0.0173	0.3481	0.0008
$T=1000$	0.7048	0.0944	0.6568	0.0080
$T=2000$	0.9526	0.1787	0.9332	− 0.0034
DGPs scenario V: models (25) and (27)				
$T=150$	0.0289	0.0072	0.0255	− 0.0086
$T=250$	0.0505	0.0144	0.0284	− 0.0010
$T=500$	0.1444	− 0.0074	0.0742	0.0038
$T=1000$	0.2387	0.0444	0.1640	0.0014
$T=2000$	0.4071	0.1593	0.3025	− 0.0096
DGPs scenario VI: models (26) and (27)				
$T=150$	0.0045	− 0.0180	− 0.0356	− 0.0024
$T=250$	0.0848	− 0.0105	0.0618	− 0.0046
$T=500$	0.1194	− 0.0082	0.1102	0.0157
$T=1000$	0.4447	− 0.0076	0.3922	0.0165
$T=2000$	0.5993	0.0258	0.5719	− 0.0004

We notice that the proposed approach provides the best classification for all the considered simulated scenarios. Moreover, the clustering accuracy improves with increasing time series length. For example, looking at the results in the scenario I, we have that with short time series $T=500$ the ARI is equal to 0.38, while with $T=2000$ it takes value of 0.88. This pattern is consistent across all the considered scenario. The validity of time-varying parameters based clustering is highlighted also by the good performances of the targeting approaches with respect to the clustering on the original time series. Furthermore, clustering based on variance leads to much more accurate results than the mean-based clustering, hence confirming the results of Cerqueti et al. (2021).

**Fig. 1:**
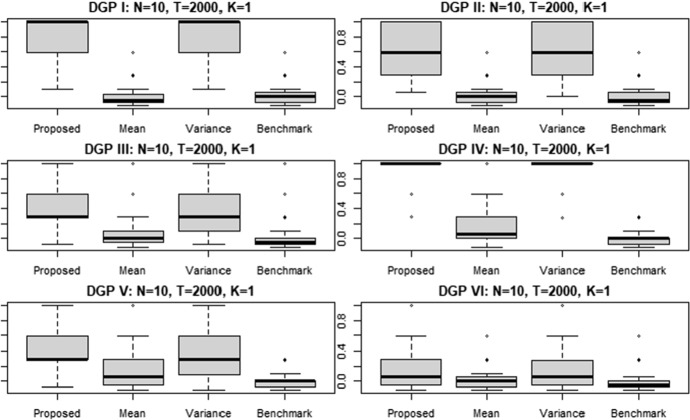
ARI of the clustering results for $N=10$ simulated univariate time series of length $T=2000$. The labels "Mean" and "Variance" indicate the clustering approaches with mean and variance targeting, respectively

Nevertheless, the values associated to the average Rand Indices vary across the simulation. The maximum value is reached in the simulated scenario II, where the proposed clustering approach provides an ARI value equal to 0.98 with $T=2000$. Similarly, in the scenario IV we obtain an ARI equal to 0.95 with very long time series. We find the lowest ARI in the scenario V, with a value equal to 0.4. However, also in this case the proposed approach outperforms all the considered alternatives. Particularly, the second best for the scenario V is represented by the clustering approach with variance targeting—which shows an ARI equal to 0.3—characterized by a much lower performance than our proposal. To explore the distribution variability of the estimated ARI, it is possible to analyze the boxplots. For example, Fig. 1 shows the ARI’s boxplots for the simulations obtained with the six alternative DGP considering a time series length of $T=2000$ and $N=10$.^3^

According to Fig. 1 we observe that the ARI obtained with the proposed approach is often characterized by lower variability and higher median value than the alternatives. Although the variability associated to the conditional mean targeting approach is generally lower that the other clustering approaches, from Fig. 1 we observe that its ARI values are often below those obtained with the proposed clustering procedure. As showed in Table 1, Fig. 1 confirms that the clustering approach with conditional variance targeting is the most competitive among the considered alternatives. The boxplots referring to the other time series length *T* are not reported here because the results are very similar to those showed in Fig. 1. Indeed, the distribution variability of the estimated ARI associated to the proposed approach is always lower than the one obtained with the conditional variance clustering approach, which is the second best. Then, although the conditional mean clustering and the benchmark based on raw data show similar or lower variability than the proposed approach, their median and average values are much lower.

The results in the case of $N=30$ and $N=60$ time series are shown in the “Appendix 1” in Tables 7 and 8, respectively. Substantially, the performance of the proposed clustering procedure is not affected by the number of statistical units in the sample. Indeed, the overperformance in terms of adjusted Rand Index achieved with the use of the proposed clustering procedure is confirmed. Furthermore, also in these cases we observe higher clustering performances with increasing time series’ length *T*. The boxplots with $T=2000$ and $N=30$ and $N=60$ are reported in Figs. 17 and 18, “Appendix 1”, showing similar results of Fig. 1. The unreported boxplots with lower time series length *T* and higher number of statistical units—i.e. $N=30$ and $N=60$—share the same patterns of those showed in the “Appendix 1”.

Then, we consider an alternative simulation scenario where multivariate time series are jointly studied. Particularly, we compare the proposed clustering algorithm based on time-varying parameters with the multi-step algorithm discussed in Košmelj (1986), Košmelj and Batagelj (1990), based on the raw time series rather than their distribution parameters.

Also in this case we consider six combination of the DGPs discussed above (24)-(27), where the *K* time series variables for a given *n*-th unit are simulated from the same DGP. For example, in the multivariate version of the scenario I, we simulate a first set of *N*/2 time series with *K* variables trough the (24) and another set of *N*/2 time series with *K* variables trough the (25). In other words, the *K* variables assume different values but are generated by the same DGP. As in the simulations with univariate time series, we consider, for each DGPs scenario, different time series’ length $T=150, 250, 500, 1000, 2000$ and different sample sizes $N=10, 30, 60$. Therefore, we end up with additional 90 alternative simulated schemes.

The results for $N=10$ are shown in Table 2.

**Table 2:** Clustering results: average Adjusted Rand Index $(N=10; \, K=2)$

Clustering approach	Proposal	Target $\mu _t$	Target $\sigma ^2_t$	Benchmark
DGPs scenario I: models (24) and (25)				
$T=150$	0.1013	− 0.0055	0.0286	0.0158
$T=250$	0.1811	0.0188	0.0040	0.0084
$T=500$	0.3959	− 0.0125	0.0389	0.0049
$T=1000$	0.8044	0.0303	0.1030	− 0.0034
$T=2000$	0.9696	0.0227	0.2177	0.0198
DGPs scenario II: models (24) and (26)				
$T=150$	0.0403	− 0.0015	0.0398	− 0.0192
$T=250$	0.0751	− 0.0101	0.0354	0.0024
$T=500$	0.1917	0.0038	0.0582	− 0.0121
$T=1000$	0.5149	0.0169	0.1457	− 0.0082
$T=2000$	0.8890	− 0.0200	0.2424	0.0004
DGPs scenario III: models (24) and (27)				
$T=150$	0.0416	− 0.0043	0.0383	0.0109
$T=250$	0.0318	0.0125	0.0343	− 0.0106
$T=500$	0.1225	0.0135	0.1135	0.0009
$T=1000$	0.3108	0.0317	0.2772	0.0037
$T=2000$	0.6456	− 0.0137	0.6189	− 0.0177
DGPs scenario IV: models (25) and (26)				
$T=150$	0.1511	0.0049	0.1385	0.0032
$T=250$	0.3157	0.0016	0.2336	− 0.0017
$T=500$	0.5516	− 0.0052	0.3833	0.0213
$T=1000$	0.8852	0.0073	0.5784	− 0.0082
$T=2000$	0.9879	− 0.0123	0.8073	0.0062
DGPs scenario V: models (25) and (27)				
$T=150$	0.0109	− 0.0030	0.0090	− 0.0059
$T=250$	0.0358	− 0.0066	0.0122	− 0.0068
$T=500$	0.1972	− 0.0082	0.1459	0.0078
$T=1000$	0.3433	− 0.0112	0.2503	0.0273
$T=2000$	0.5914	0.0174	0.4955	− 0.0070
DGPs scenario VI: models (26) and (27)				
$T=150$	0.0516	0.0094	0.0127	0.0111
$T=250$	0.0776	0.0091	0.0222	0.0348
$T=500$	0.2048	0.0014	0.1407	0.0023
$T=1000$	0.3734	− 0.0020	0.2422	− 0.0255
$T=2000$	0.5842	− 0.0102	0.3888	0.0121

The results in terms of average ARI are, compared to the benchmark approach, outstanding especially with long time series’ length *T*. For example, in the scenario I of Table 2 the average ARI is equal to 0.96 for the proposed approach, while the benchmark provides a random partition with an ARI close to 0. Similarly good results are achieved with the scenario IV, where the ARI associated to the proposed clustering approach is equal to 0.98. Moreover, for these simulations we have that the lowest average ARIs associated to the developed clustering procedure are always close to 0.6 for long time series. For example, in the simulated scenario V it is equal to 0.6 versus the value of 0 of the benchmark.

With shorter time series the results are still good. For example, in the scenario I, we obtain an ARI equal to 0.8 with $T=1000$ and 0.4 with $T=500$. Unfortunately, not all the simulated scenario show high performances with very short time series $T=250$ and $T=150$. The results obtained with $T=150$ are very close to those with $T=250$. The best result is achieved with the scenario IV, where the average ARI is equal to 0.3. However, in many cases the average ARI is similar to the benchmark. Therefore, these results confirm that the proposed clustering approach works particularly well in presence of longer time series. This can be explained by the very good performances of the ACF-based distance with long time series data. Conversely, it is known that the performances of the ACF-based tend to be less accurate with short time series.

From these simulations it is evident that the benchmark model is associated to an always very low adjusted Rand Index. The so high performances of the proposed approach can be justified by the DGP, characterized by time variation in the distribution parameters. With the right specification of the DGP, the results in terms of clustering quality resulting by the use of time-varying parameters are very satisfactory.

**Fig. 2:**
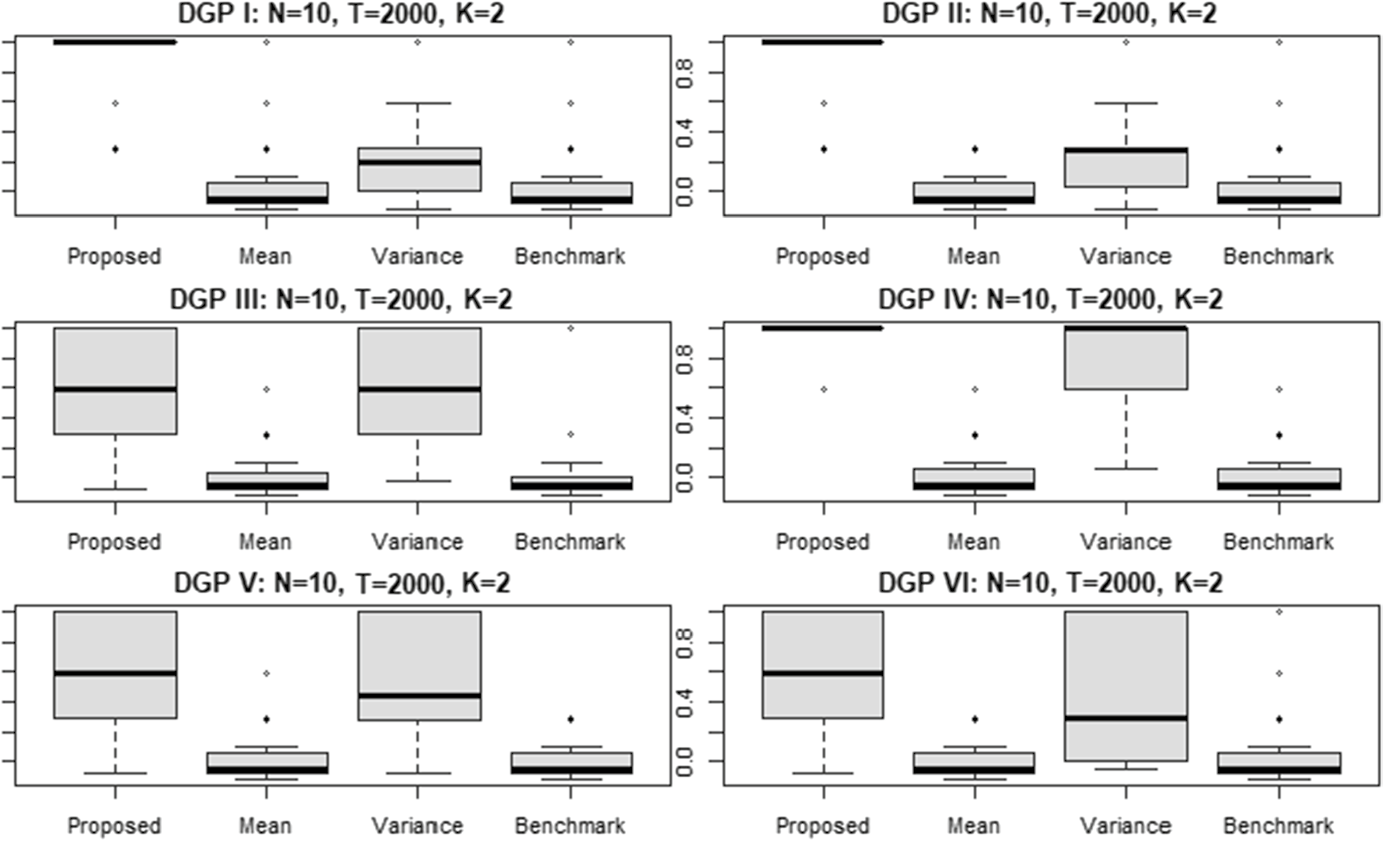
ARI of the clustering results for $N=10$ simulated multivariate time series $K=2$ each of length $T=2000$. The labels "Mean" and "Variance" indicate the clustering approach with mean and variance targeting, respectively

As in the univariate case, for exploring the distribution variability of the estimated ARI, it is possible to analyze the boxplots. For example, Fig. 2 shows the ARI’s boxplots for the simulations obtained with the six alternative DGP considering $K=2$ time series length of $T=2000$.^4^

The proposed clustering procedure performs particularly well in the simulated scenarios with DGP I, DPG II and DPG IV. Indeed, in these cases we have that the variability of the estimated ARI is very low also compared with the conditional variance targeting approach, which represents the second best. The median ARI for the proposed procedure equals to the maximum value of 1 in such simulated scenarios. Quite similar conclusions can be derived from the other scenarios. Furthermore, we observe that the proposed clustering procedure is characterized by lower variability and higher median values than the alternatives, although the variability of the results under the DGPs III, V and VI is higher than in the DGPs I, II and IV. These results confirm those of univariate time series. As in the univariate case, the boxplots referring to simulated scenarion with other time series length *T* are not reported. Indeed, the obtained results in these unreported cases are close to those showed in Fig. 2. Indeed, considering the ARI obtained with the proposed clustering approach, we find a variability that is always lower (in some simulated scenarios it is very similar) to the one associated with the ARI of the conditional variance clustering approach, which is also in the multivariate case the best among the considered alternatives. The ARI associated to the other two alternative clustering approaches—i.e. conditional mean and raw data-based—in general show the same variabilty of our procedure, but with much lower median and average values. Although in some simulated scenarios the conditional mean clustering shows lower variability (e.g. with DGP IV and $T=150$ or with DGP V and $T=250$), such lower variability comes at a cost: lower clustering performances. Therefore, also the analysis of the boxplots shows that the proposed procedure outperforms the considered alternatives.

In the end, we evaluate how the performances change with increasing sample size *N*. The results of simulations with $N=30$ and $N=60$ are shown in Tables 9 and 10, in the “Appendix 1”.

As in the univariate setting, also for multivariate time series the number of statistical units to be clustered does not affect the clustering quality. Tables 9 and 10 confirm the very good performances of the proposed clustering approach with very long time series’ length. Scenario I and IV provide the best results, with average ARI equal to 0.97 and 0.99, respectively. The benchmark model is characterized by very poor performances, confirming that when the distribution parameters change over time a clustering approach that considers the raw time series should not be used. Finally, also with increasing *N*, we observe very high clustering performances with medium and long time series, whereas the good performances for short time series are not robust across all the simulations.

The boxplots with $T=2000$ and $N=30$ and $N=60$ are reported in Figs. 19 and 20 in the “Appendix 1”. The results are similar to those showed in Fig. 2. The unreported boxplots, associated to simulated scenarios with lower time series length *T* and $N=30$ and $N=60$, share the same patterns of those showed in the “Appendix 1”. Overall, the boxplots with different number of statistical units, i.e. $N=30$ and $N=60$, do not differ from the case $N=10$.

## Application to air quality time series data

In what follows we show an application of the proposed clustering procedure to environmental time series with the aim of identifying groups of cities characterized by the same levels of air quality.

### Data

Air quality monitoring is conducted by means of stations that measure the content of atmospheric pollutants and weather conditions. By aggregating data, it is possible to obtain the air quality patterns for a given region or city. Air quality is also related to many of the United Nations Sustainable Development Goals. For example, the development of policies aimed at reducing the emission of pollutants in the air are directly related with climate mitigation targets, access to clean energy services, waste management, and other aspects of socio-economic development (Lu et al. 2015; Rafaj et al. 2018).

The application with real data is conducted on the most important cities in India^5^. In particular, we considered daily air quality time series about Particulate Matter (PM) with values expressed in micron, PM2.5 and PM10, in the period 1$^{th}$ January 2020– 1$^{th}$ June 2020. The data at city level are aggregated considering many stations placed within each city^6^. The final sample is characterized by $N=15$ units (i.e. the cities) observed for $T=182$ time periods.

**Fig. 3:**
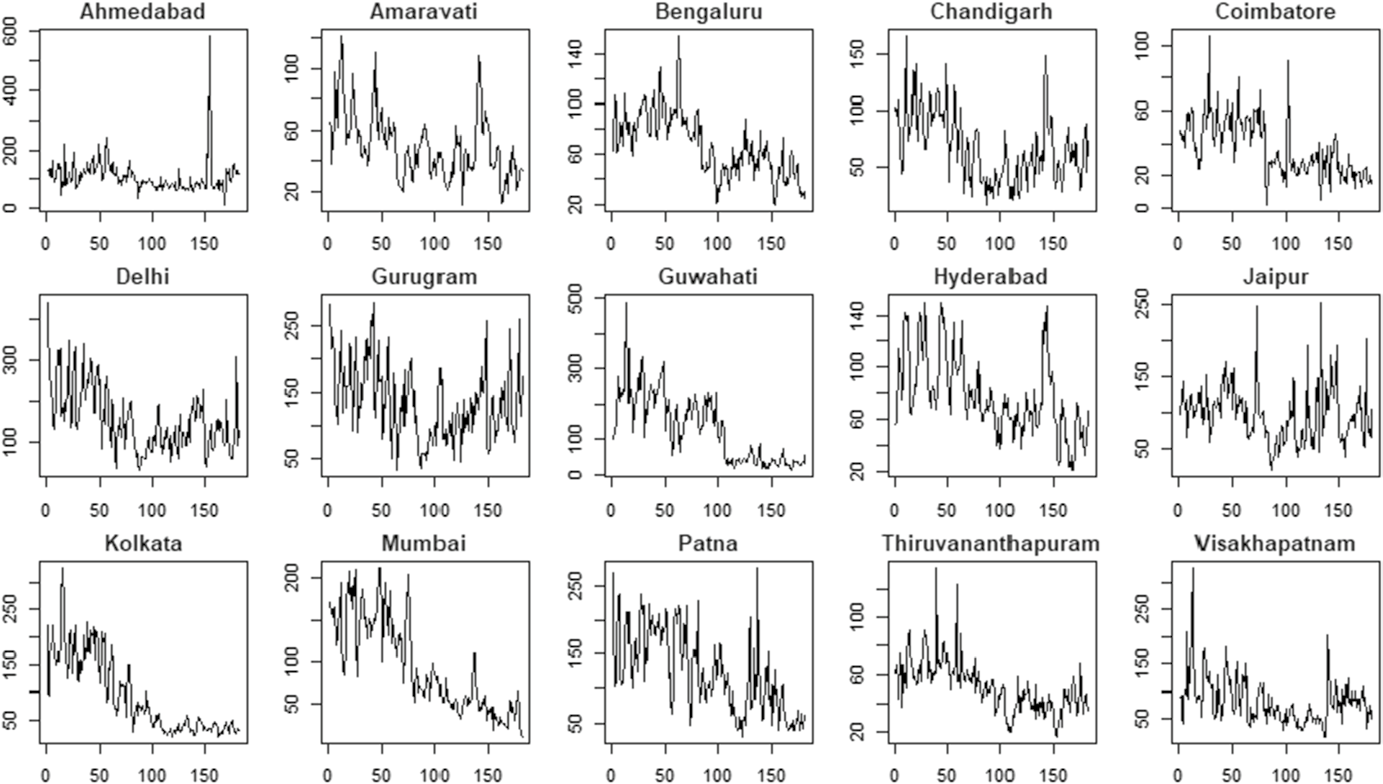
PM2.5 indicator—time series

**Fig. 4:**
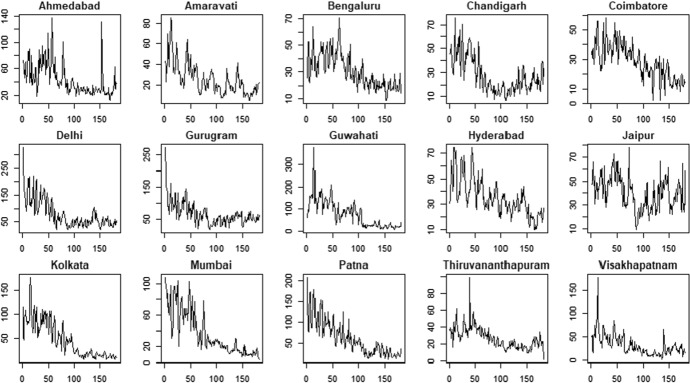
PM10 indicator—time series

The air pollution time series are shown in Figs. 3 (PM2.5) and Fig. 4 (PM10).

The PM2.5 and PM10 time series present some similarities in their patterns for all the cities. For example, we observe that most of the cities show a reduction in air pollution during the period 03/2020–06/2020 according to both the variables. However, there are also significant differences among the cities: some cities are characterized by negative trends (e.g. Kolkata and Mumbai) whereas some others show more stable patterns (e.g. Gurugram and Jaipur).

The presence of deterministic trends in the air pollution time series indicates that the underling processes are not stationary. As discussed in Blasques et al. (2022), stationarity of the observed time series is needed to ensure consistency of maximum likelihood estimator in the case of model misspecification for the GAS processes. For this reason, we prefer to analyze the air pollution’s rate of changes which have the same information for the problem at hand, i.e. clustering cities with same levels of air quality.

### Results with Gaussian density

**Fig. 5:**
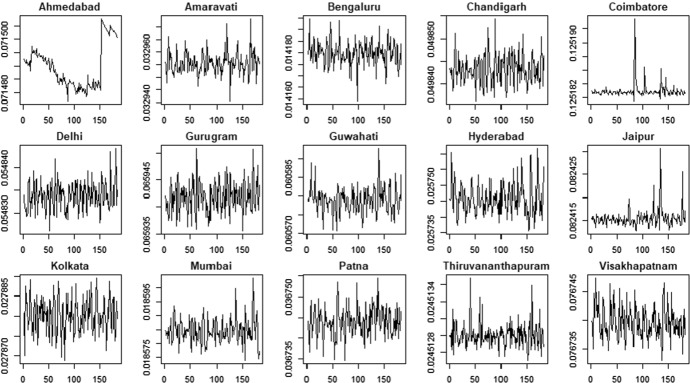
PM2.5 indicator—time-varying mean

**Fig. 6:**
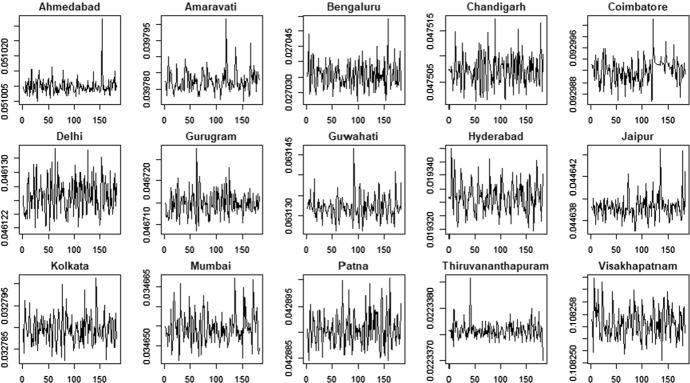
PM10 indicator—time-varying mean

**Fig. 7:**
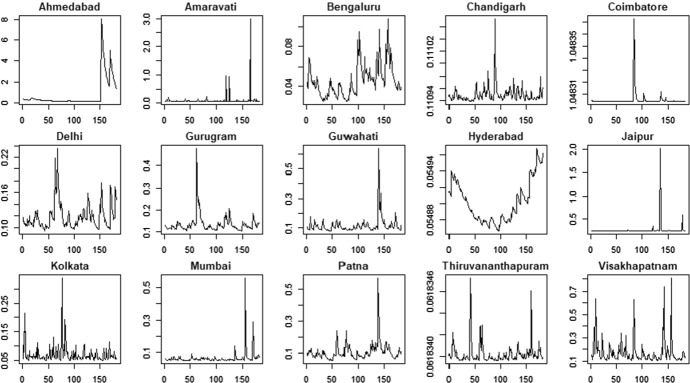
PM2.5 indicator—time-varying variance

**Fig. 8:**
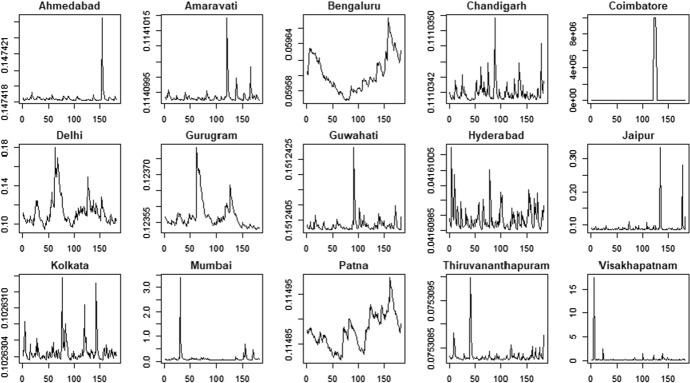
PM10 indicator—time-varying variance

Figures 5 and 6 show the pattern of the estimated time-varying mean under the hypothesis of Gaussian distribution, while Figs. 7 and 8 show the time-varying variance.

The time-varying parameters provide some useful information about the pattern of air pollution. For example, considering the PM2.5 variable, Coimbatore and Jaipur show a lower level of variability in the conditional mean that fluctuates around a constant value with some spikes associated to days with very low air quality levels. In contrast, Gurugram and Kolkata are characterized by high variability in the conditional mean. These results are confirmed by the analysis of conditional variances shown in Fig. 7, with cities like Coimbatore and Jaipur characterized by quite flat conditional variances and others, like Gurugram and Kolkata, that show the typical pattern of conditionally heteroskedastic processes. The city of Hyderabad, instead, presents a very peculiar pattern for the conditional variance, which differs from the variances observed in the other cities. Considering the PM10 time series (Fig. 8) we also observe clear differences in the time-varying parameters. Coimbatore and Visakhapatnam are characterized by conditional means with low variability, reflecting the quite flat structure of conditional variances. Also in the case of PM10, we recognize that Hyderabad has a very peculiar pattern of the conditional variance. Therefore, we suspect that this city can be an outlier.

**Fig. 9:**
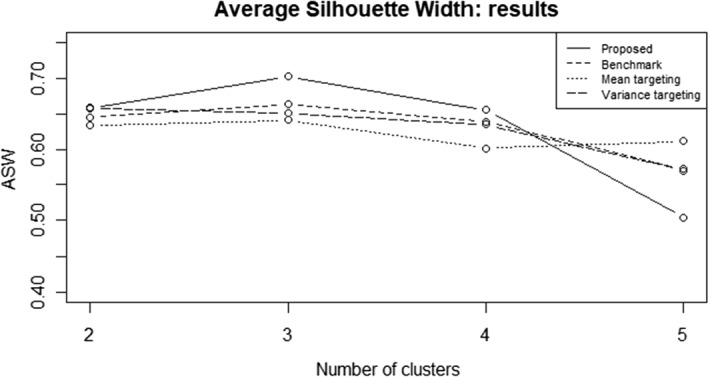
Average Silhouette Width: proposed approach (solid line) comparison with benchmark (dashed line), mean targeting (dotted line) and variance targeting (long dash line)

We compared the partition obtained by the use of the proposed clustering approach with the one based on the raw time series and the two clustering approaches involving parameter targeting by means of the Average Silhouette Width (ASW) criterion. The results are shown in Fig. 9.

In Fig. 9, the solid line represents the ASW of the proposed clustering procedure based on time-varying parameters, whereas the dashed line shows the ASW values associated to the benchmark for different number of clusters. We note that both the procedures define as optimal number of clusters $C=3$, but our procedure provides a better partition (the line associated with our procedure is always above those of the benchmarks).

The resulting partitions are shown in Table 3.

**Table 3:** Clustering results under Gaussian distribution

City	Clustering approach			
	Benchmark	Proposed approach	Mean target	Variance target
Ahmedabad	1	1	1	1
Amaravati	1	1	2	2
Bengaluru	1	2	3	1
Chandigarh	2	1	2	2
Coimbatore	1	1	3	2
Delhi	3	2	2	2
Gurugram	3	2	2	2
Guwahati	1	1	3	2
Hyderabad	2	3	2	3
Jaipur	3	1	2	2
Kolkata	3	1	2	2
Mumbai	3	1	2	2
Patna	1	2	3	3
Thiruvananthapuram	2	1	2	2
Visakhapatnam	2	1	2	2

Although the groups’ composition differs according to the two considered clustering procedures, some similarities can be highlighted. For example, some cities are clustered together according to the considered approaches. Examples are the cities of Ahmedabad, Amaravati and Coimbatore but also Delhi and Gurugram. This means that the same levels of air quality characterize these cities. However, despite these similarities, the clustering results are different. First of all, our procedure highlights the presence of an outlier, identified as the Hyderabad city, which is the only unit belonging to cluster 3. On the contrary, no outliers are identified by the benchmark clustering approach based on raw data and with variance targeting, while the conditional mean targeting approach considers the city of Ahmedabad as outlier.

As a consequence, also the groups’ size is different. Indeed, the benchmark clustering algorithm based on raw data assigns the cities in the similarly sized clusters, with six cities placed in cluster 1, four cities in cluster 2 and five cities to cluster 3. The mean targeting approach assigns most cities in cluster 2 and four cities in cluster 3. The variance targeting does not highlight any outlier, placing most cities in cluster 2 and two cities in cluster 1 and cluster 3. Differently, our procedure highlights that most Indian cities are placed in cluster 1 (ten units), and a residual part of them is placed in cluster 2 (four units). By looking at the average values of air quality within the clusters (see Table 4), we suppose that the resulting classification could imply some differences in environmental policies.

**Table 4:** Average values within clusters—proposed clustering procedure with Guassian density

Panel A: PM2.5				
*Cluster 1:*				
Ahmedabad	Amaravati	Chandigarh	Coimbatore	Guwahati
107.66552	50.08341	66.86610	37.27478	126.52324
Jaipur	Kolkata	Mumbai	Thiruvananthapuram	Visakhapatnam
97.12846	93.44082	94.24385	51.39780	82.46033
*Cluster 2:*				
Bengaluru	Delhi	Gurugram	Patna	
67.01093	155.23841	133.20791	122.85110	
*Cluster 3:*				
Hyderabad				
78.01857				

Panel B: PM10				
*Cluster 1:*				
Ahmedabad	Amaravati	Chandigarh	Coimbatore	Guwahati
43.33951	25.81865	27.50143	28.40712	74.17467
Jaipur	Kolkata	Mumbai	Thiruvananthapuram	Visakhapatnam
40.15648	46.60440	36.22363	26.79978	31.57588
*Cluster 2:*				
Bengaluru	Delhi	Gurugram	Patna	
30.46412	78.71456	65.75945	60.48154	
*Cluster 3:*				
Hyderabad				
35.06434				

The proposed clustering procedure allows us to identify the cities characterized by low air quality, i.e. high levels of the PM2.5 and PM10 indicators. More precisely, the cities belonging to cluster 2 show the highest levels of particular matter (PM) in the air. Conversely, the cities in cluster 1 show lower average values. Therefore, cluster 1 includes cities with better air quality. Hyderabad is considered an outlier because of the conditional variance patterns in the air pollution indicators, as shown in Figs. 7 and 8. These results suggest improving air quality in cities belonging to cluster 2, which should be more closely monitored.

### Results with Generalized-t density

To evaluate the impact of the modelling hypothesis on the final results, we also assessed the clusters’ change under an alternative distributional assumption. In the case of environmental time series, which are heavy-tailed (e.g. see Muller 2016; Williams et al. 2020), it can be more appropriate to use a conditional non-Gaussian model. Thanks to the flexibility of the GAS model, the proposed clustering procedure can be extended to the case of non-Gaussian distributions. In Sect. 2.4 we have introduced the case of Generalized-t distribution-based clustering procedure. Starting from the same dataset discussed in Sect. 4.1, in what follows we apply the proposed clustering procedure under the Generalized-t distributional assumption.

**Fig. 10:**
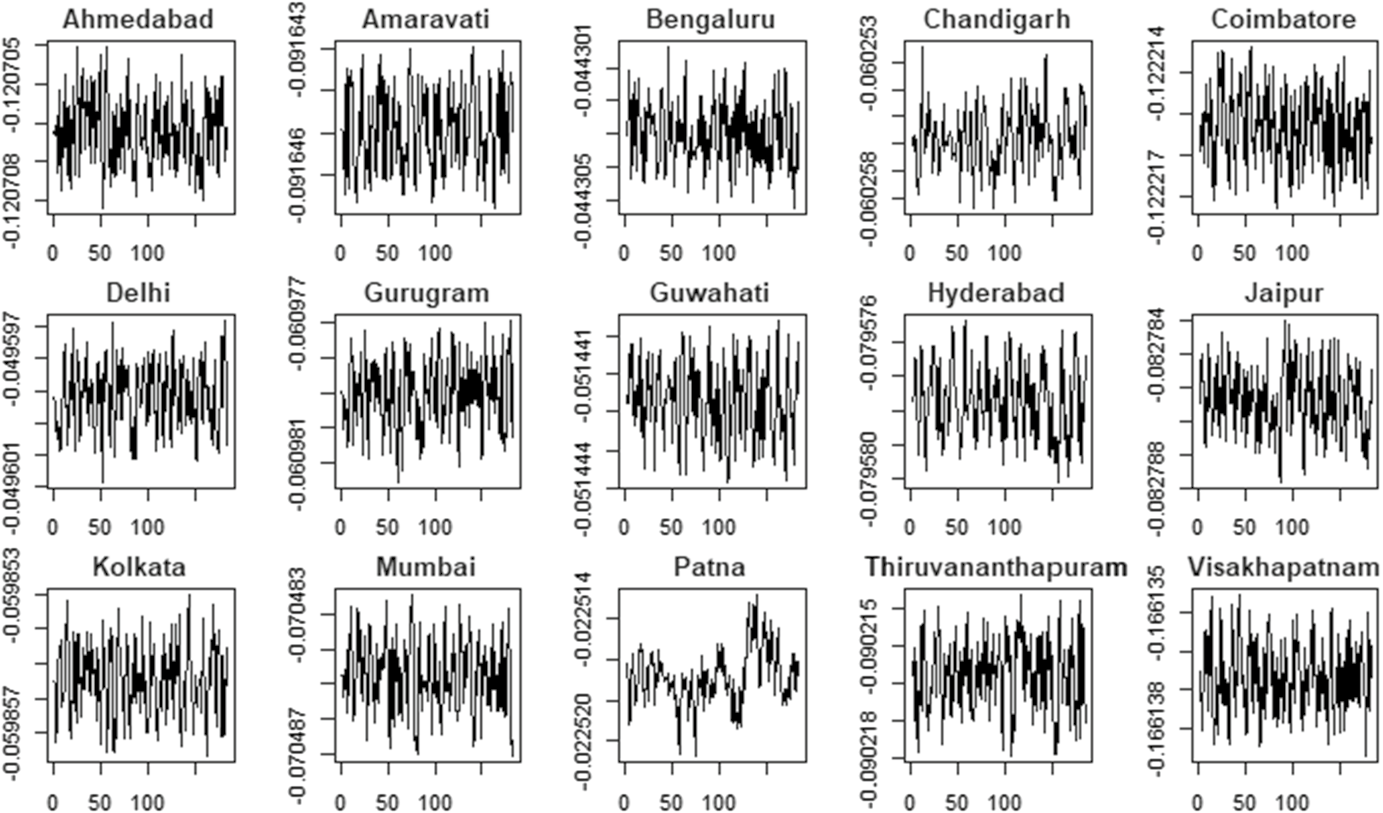
PM2.5 indicator—time-varying location

**Fig. 11:**
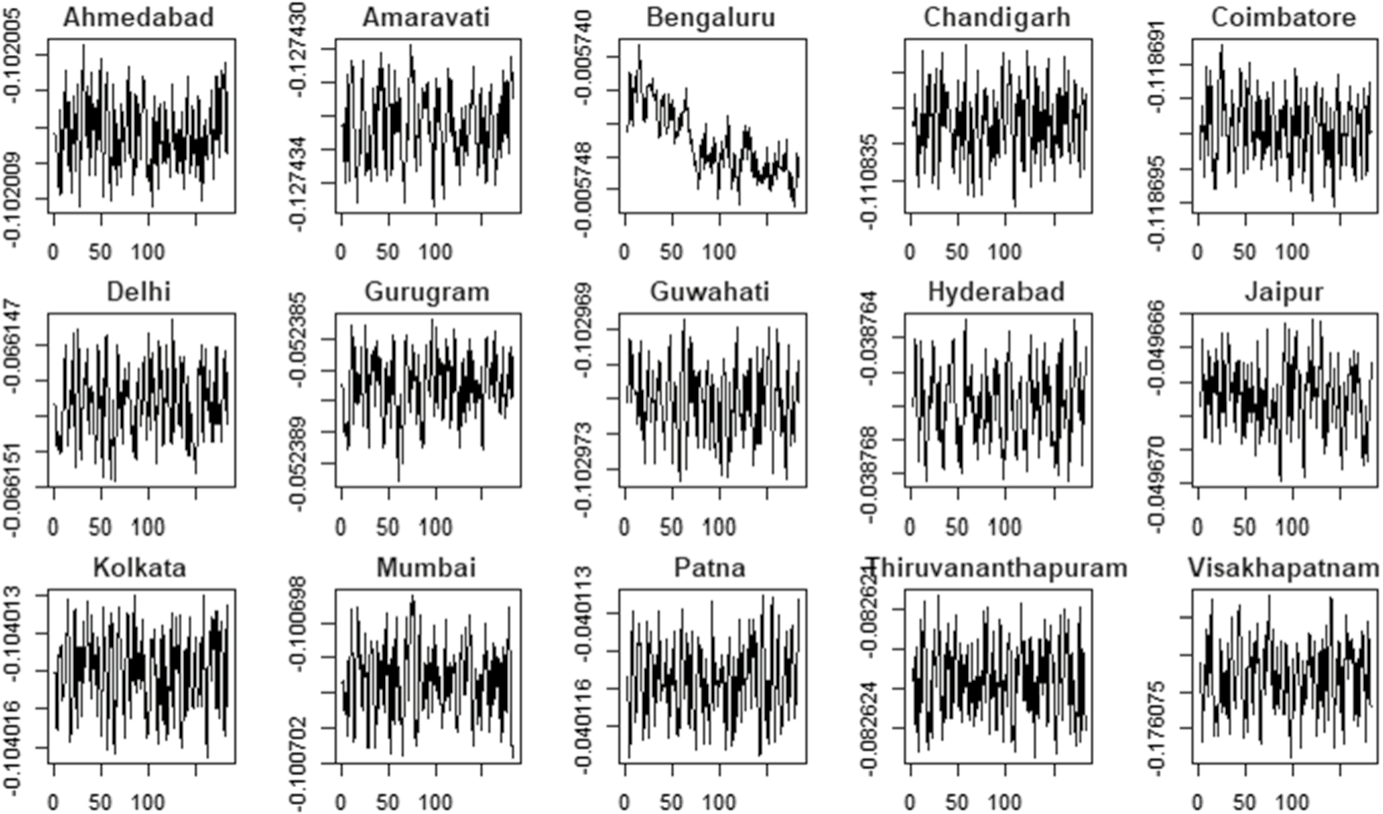
PM10 indicator—time-varying location

**Fig. 12:**
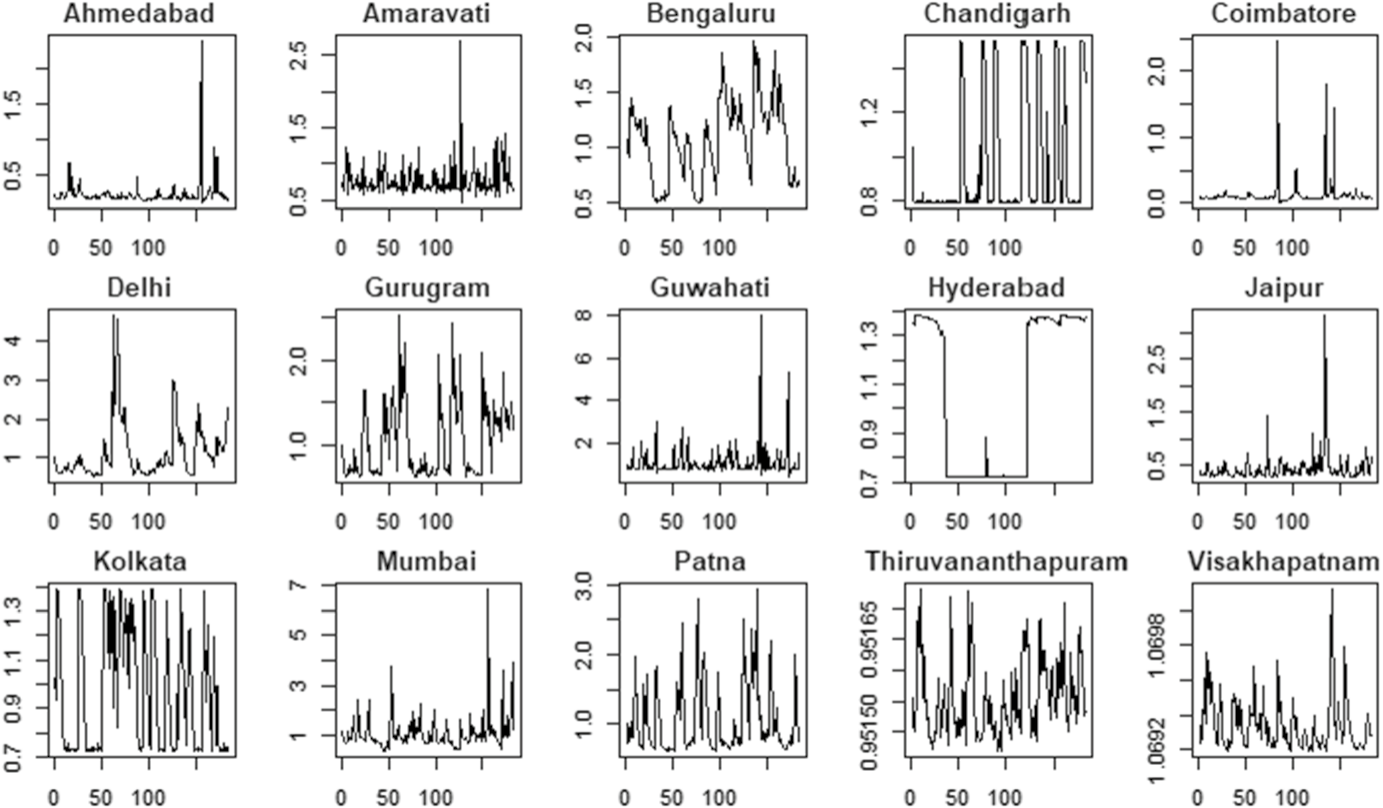
PM2.5 indicator—time-varying scale

**Fig. 13:**
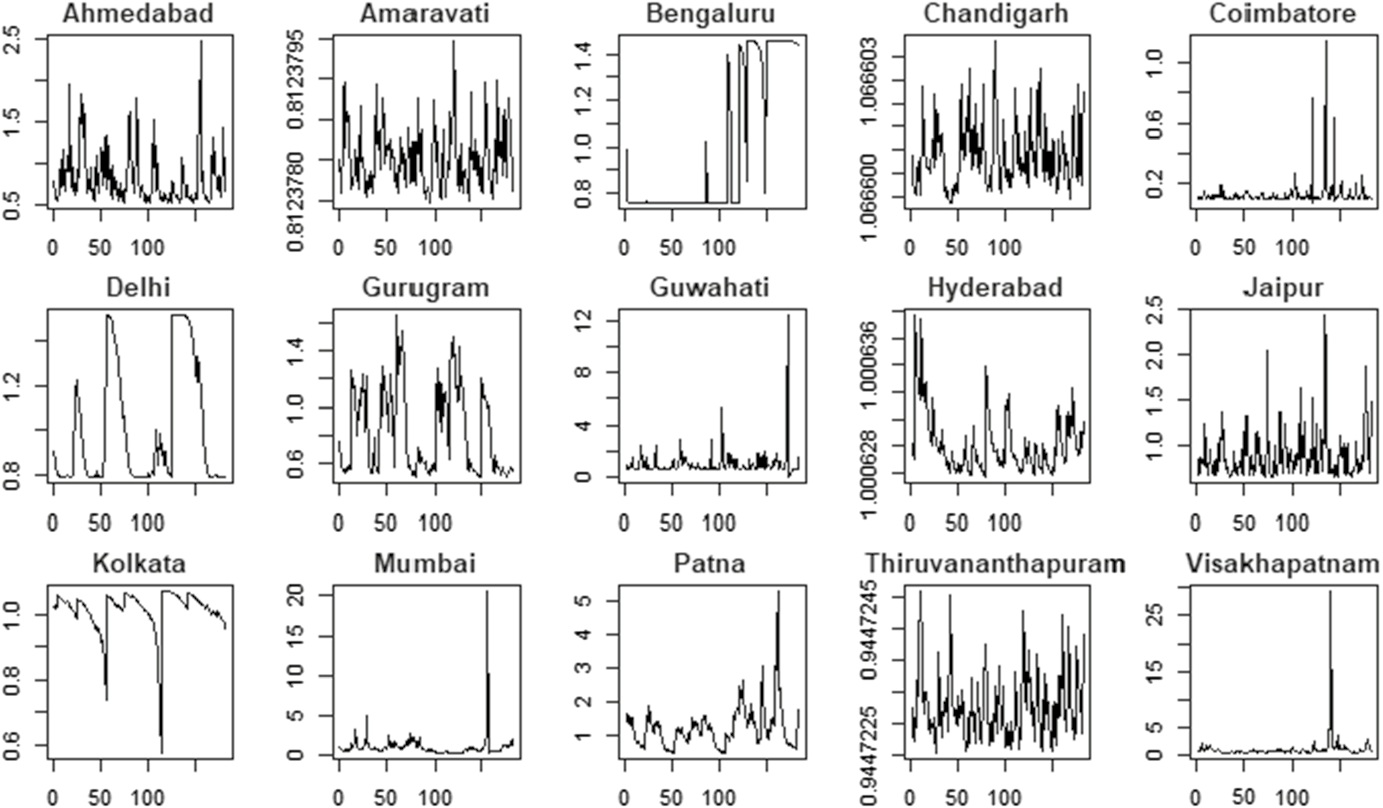
PM10 indicator—time-varying scale

**Fig. 14:**
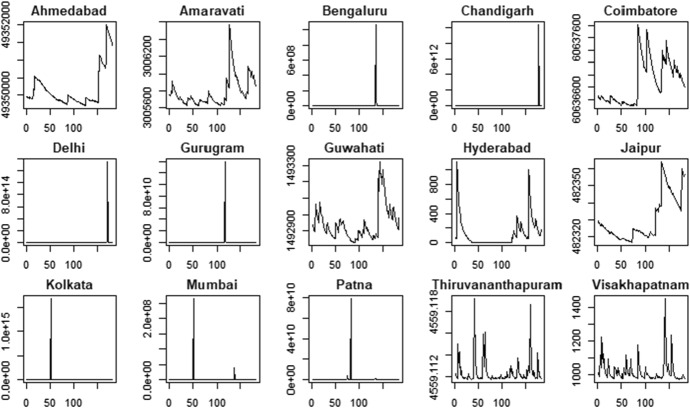
PM2.5 indicator—time-varying shape

**Fig. 15:**
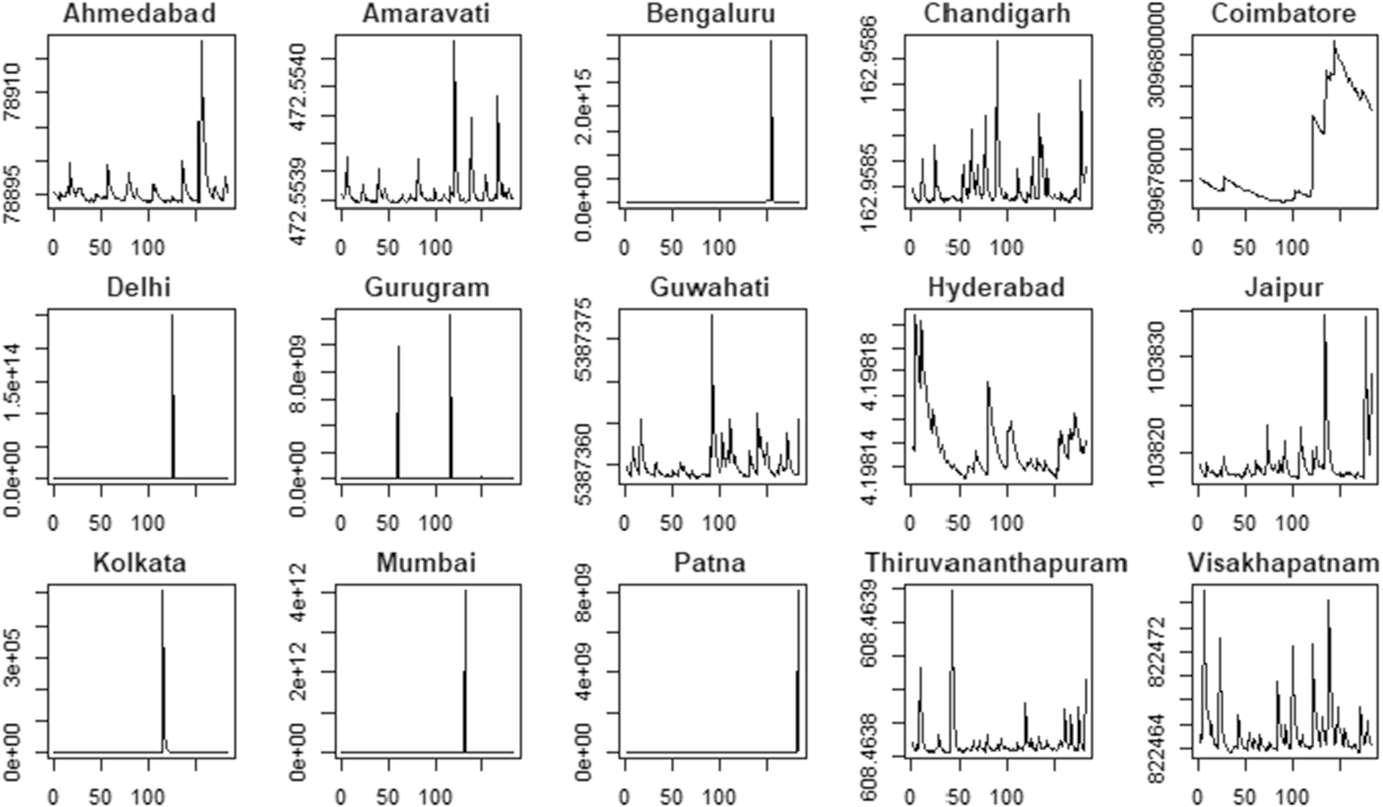
PM10 indicator—time-varying shape

Figures 10 and 11 show the time series of the estimated time-varying location under the hypothesis of Generalized-t distribution, Figs. 12 and 13 show the estimated time-varying scale and Figs. 14 and 15 show the time-varying shape for both PM2.5 and PM10 time series.

The time-varying location parameters, shown in Figs. 10 and 11, are characterized by fluctuations around a constant long-run value. Two exceptions are the time-varying location of the variable PM2.5 and PM10 related to the city of Patna and Bengalauru, which show a positive trend in the first case and a negative one in the second case. The time-varying scale parameters are shown in Figs. 12 and 13.Moreover, the Hyderabad and Bengalauru cities show time-varying scale parameters of the PM2.5 and PM10 which are very different from those of the other cities. Therefore, the Bengalauru city could be considered as a possible outlier in terms of both location and scale. In the end, the time-varying shape parameters are showed in Figs. 14 and 15. Time-varying shape parameters are interestingly characterized by stationary patterns followed by a large peak. The city of Gurugam is characterized by two large peaks in the variable PM10.

**Fig. 16:**
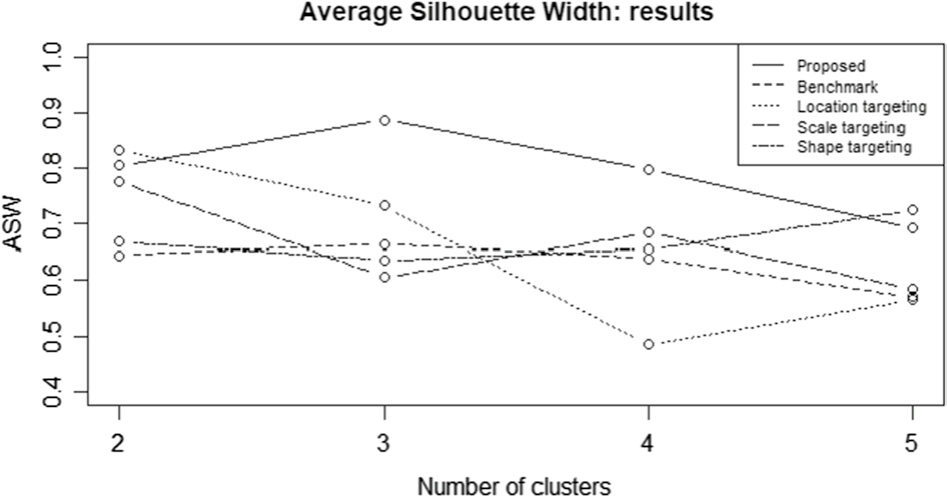
Average Silhouette Width: proposed approach (solid line) comparison with benchmark (dashed line), location targeting (dotted line) and scale targeting (long dash line)

**Fig. 17:**
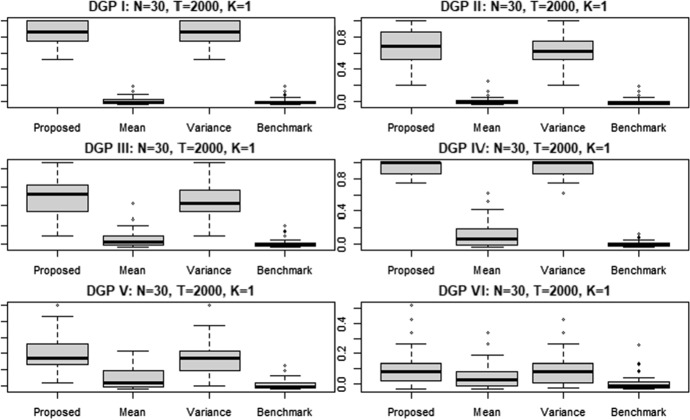
ARI of the clustering results for $N=30$ simulated univariate time series of length $T=2000$

**Fig. 18:**
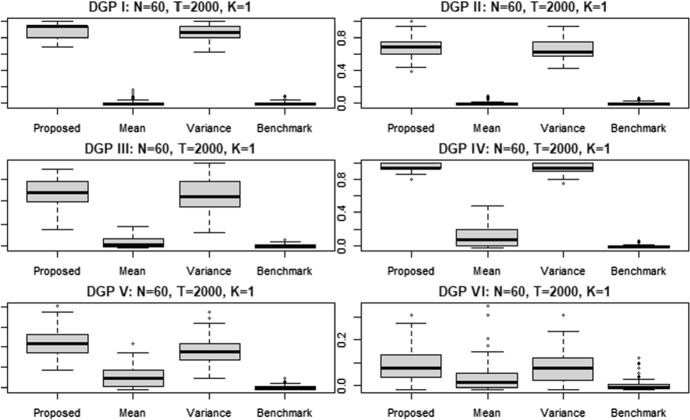
ARI of the clustering results for $N=60$ simulated univariate time series of length $T=2000$

**Fig. 19:**
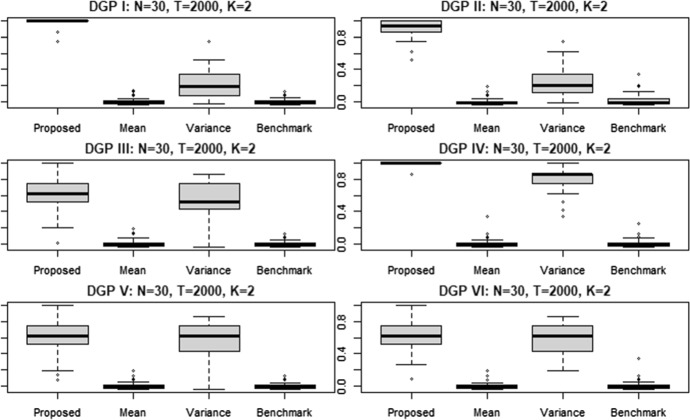
ARI of the clustering results for $N=30$ simulated multivariate time series $K=2$ each of length $T=2000$

**Fig. 20:**
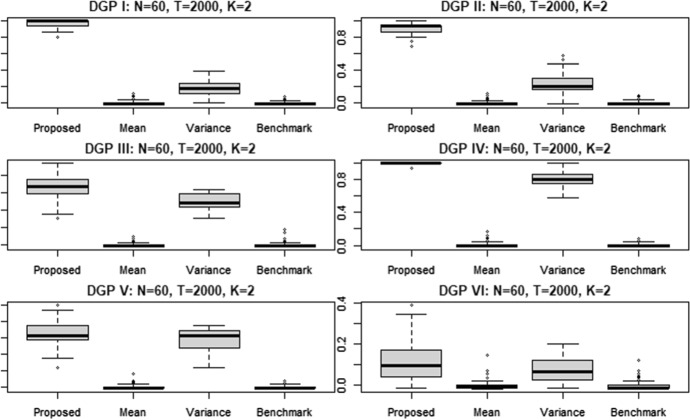
ARI of the clustering results for $N=60$ simulated multivariate time series $K=2$ each of length $T=2000$

We compared the partition obtained by the use of the proposed clustering approach with the selected benchmarks by means of the Average Silhouette Width (ASW) criterion. The results are shown in Fig. 16.

In terms of ASW, the proposed approach achieves the highest value, about 0.9 with $C=3$ clusters, among the alternatives. We note that the ASW curve associated with the proposed clustering procedure is always above those of the alternative approaches. This suggests that it provides a better partition.

Some differences and similarities with the results obtained under a Gaussian distribution assumption can be highlighted. For example, as in the Gaussian case, the proposed clustering procedure maximizes the ASW with $C=3$. This suggests that a partition with three clusters is probably the most appropriate for the analyzed dataset. However, the benchmark approaches, under the Generalized-t distributional assumption, indicate the presence of $C=2$ clusters, in the case of location and scale targeting approaches, $C=5$ for the shape targeting approach. The raw data-based approach also suggests the presence of $C=3$ clusters.

It is important to highlight that, under the Generalized-t assumption, all the clustering algorithms improve their performances compared to those with the Gaussian distribution. This suggests that the Generalized-t distribution better describes the considered environmental time series.

The resulting partitions are shown in Table 5.

**Table 5:** Clustering results under Generalized-t distribution

City	Clustering approach				
	Benchmark	Proposed approach	Location target	Scale target	Shape target
Ahmedabad	1	1	1	1	1
Amaravati	1	1	1	1	1
Bengaluru	1	2	2	2	2
Chandigarh	2	1	1	1	3
Coimbatore	1	1	1	1	4
Delhi	3	3	1	1	3
Gurugram	3	1	1	1	4
Guwahati	1	1	1	1	4
Hyderabad	2	1	1	2	5
Jaipur	3	1	1	1	4
Kolkata	3	3	1	1	3
Mumbai	3	1	1	1	4
Patna	1	1	2	1	3
Thiruvananthapuram	2	1	1	1	1
Visakhapatnam	2	1	1	1	1

We note that, in the case of conditional scale targeting, most cities are grouped together with the exception of Hyderabad and Bengaluru. This can be due to the time patterns of their conditional scale parameters for PM2.5 (Hyderabad) and PM10 (Bengaluru). Looking at the partition obtained with the conditional location targeting, Bengaluru and Patna are placed in the cluster 2 because of peculiar patterns of PM10 (Bengaluru) and PM2.5 (Patna). The conditional shape targeting provides a partition with two outliers, Bengalauru and Hyderabad. The proposed clustering procedure provides a partition taking jointly into account all the time-varying parameters. Therefore, it shows a unique outlier in the sample: the city of Bengaluru, with location (PM10) and scale (PM10) very different from the other cities.

Then, we consider the average values of air quality variables PM2.5 and PM10 within the clusters. Table 6 highlights interesting differences among the groups obtained with the proposed approach.

**Table 6:** Average values within clusters—proposed clustering procedure with Generalized-t density

Panel A: PM2.5			
*Cluster 1:*			
Ahmedabad	Amaravati	Chandigarh	Coimbatore
107.66552	50.08341	66.86610	37.27478
Gurugram	Guwahati	Hyderabad	Jaipur
133.20791	126.52324	78.01857	97.12846
Mumbai	Patna	Thiruvananthapuram	Visakhapatnam
94.24385	122.85110	51.39780	82.46033
*Cluster 2:*			
Bengaluru			
67.01093			
*Cluster 3:*			
Delhi	Kolkata		
155.23841	93.44082		

Panel B: PM10			
*Cluster 1:*			
Ahmedabad	Amaravati	Chandigarh	Coimbatore
43.33951	25.81865	27.50143	28.40712
Gurugram	Guwahati	Hyderabad	Jaipur
65.75945	74.17467	35.06434	40.15648
Mumbai	Patna	Thiruvananthapuram	Visakhapatnam
36.22363	60.48154	26.79978	31.57588
*Cluster 2:*			
Bengaluru			
30.46412			
*Cluster 3:*			
Delhi	Kolkata		
78.71456	46.60440		

According to PM2.5, we observe that cluster 3 includes cities with very high average values, whereas cluster 1 is more heterogeneous in its composition. The city of Bengalauru has a relatively low value of PM2.5, which is close to the first quartile of the distribution. In the cluster 3 we have two cities with high PM2.5 average values. Delhi is the city with the maximum PM2.5 average value, while Kolkata has a value close to the third quartile of the distribution. Similar patterns can be find looking at the average values of PM10 variable.

## Final remarks

Clustering time series according to their distribution parameters is a widely explored topic. In this framework, some recent contributions consider time variation in the distribution parameters, but only in the case of univariate time series. This paper provides a clustering procedure based on time-varying parameters for multivariate time series.

Clustering multivariate time series with time-varying parameters is not straightforward because the data structure is a 4D tensor. The four dimensions are: (1) the statistical units, (2) the time, (3) the variables, and (4) the distribution parameters. In the proposed multiway clustering procedure, we adopt a multi-step approach where, firstly, a dissimilarity matrix, for each 3D tensor included in the 4D tensor, is computed. Then, starting from each distance matrix, the consensus matrix is computed by the DISTATIS algorithm Abdi et al. (2005). The final partition is obtained by using this distance matrix as input of the PAM algorithm.

An extensive simulation study, conducted considering both different time series lengths, sample sizes and number of variables, compares the performance of the proposed clustering procedure with the one of a standard multi-step clustering procedure for 3D tensors applied to the raw time series. For all the considered scenarios, the proposed approach outperforms the alternatives. The usefulness of the proposed clustering is discussed through an application to environmental time series about air quality. As a further support to the validity of our procedure, we notice that the proposed procedure performs in partitioning the considered dataset, although the time series considered in the application are not very long. For this aim, we compare the clusters obtained using the proposed approach with those obtained considering a standard multi-step clustering approach for multiway data.

Some future research developments can be highlighted. Firstly, we notice that the procedure developed in the paper can be used for clustering any 4D tensor. Therefore, it can also be adopted for clustering 4D tensors that do not include time-varying parameters. Secondly, we also highlight that the proposed approach could be extended to account for co-moments, such as covariance, coskewenss and cokurtosis. This aspect is relevant when the time series show a cross-dependence structure in higher moments of the distribution. A third line of future research lies on the parameters’ distribution weighting. Indeed, in the present paper we implicitly assign equal weights to the different time-varying parameters. However, as shown in Cerqueti et al. (2022), it could be interesting to assign different weights to the distribution parameters and search for the optimal weights. This aspect should be taken into account in future studies. In the end, the proposed clustering approach can be extended to include spatial dependence in the data. Spatial dependence arise when dealing with statistical units that are observed over both time and space, such provinces, cities or countries. Therefore, the extension of the proposed clustering procedure to spatio-temporal setting represents another interesting future research line.

## Appendix 1: Simulation study: more results

See Figs. 17, 18, 19, 20 and Tables 7, 8, 9, 10.

**Table 7:** Clustering results: average Adjusted Rand Index $(N=30; \, K=1)$

Clustering approach	Proposal	Target $\mu _t$	Target $\sigma ^2_t$	Benchmark
DGPs scenario I: models (24) and (25)				
$T=150$	0.0606	0.0057	0.0406	0.0009
$T=250$	0.1350	− 0.0008	0.0975	− 0.0058
$T=500$	0.3412	0.0088	0.2895	0.0117
$T=1000$	0.6336	− 0.0066	0.5551	0.0016
$T=2000$	0.8738	0.0053	0.8380	− 0.0025
DGPs scenario II: models (24) and (26)				
$T=150$	0.0161	0.0092	0.0142	0.0003
$T=250$	0.0202	0.0161	0.0401	0.0271
$T=500$	0.05208	0.01920	0.04736	− 0.01288
$T=1000$	0.20224	0.05344	0.16360	0.05384
$T=2000$	0.80688	0.43688	0.74232	0.02304
DGPs scenario III: models (24) and (27)				
$T=150$	0.0123	− 0.0012	0.0092	0.0008
$T=250$	0.0317	0.0123	0.0329	− 0.0028
$T=500$	0.0887	0.0070	0.0715	− 0.0016
$T=1000$	0.1990	0.0236	0.1864	0.0039
$T=2000$	0.4789	0.0589	0.4482	− 0.0032
DGPs scenario IV: models (25) and (26)				
$T=150$	0.0936	0.0017	0.0641	− 0.0026
$T=250$	0.1952	0.0064	0.1193	− 0.0009
$T=500$	0.3942	0.0213	0.3437	− 0.0043
$T=1000$	0.7189	0.0344	0.6739	− 0.0016
$T=2000$	0.9523	0.1117	0.9266	0.0002
DGPs scenario V: models (25) and (27)				
$T=150$	0.0223	0.0024	0.0084	0.0034
$T=250$	0.0702	− 0.0025	0.0463	− 0.0046
$T=500$	0.1153	0.0295	0.0772	0.0039
$T=1000$	0.2593	0.0364	0.1843	− 0.0071
$T=2000$	0.4034	0.0934	0.3228	0.0075
DGPs scenario VI: models (26) and (27)				
$T=150$	0.0079	0.0047	0.0038	− 0.0068
$T=250$	0.0474	0.0005	0.0395	0.0036
$T=500$	0.1500	− 0.0043	0.1190	0.0005
$T=1000$	0.3960	0.0032	0.3667	0.0035
$T=2000$	0.6601	− 0.0053	0.6498	− 0.0042

**Table 8:** Clustering results: average Adjusted Rand Index $(N=60; \, K=1)$

Clustering approach	Proposal	Target $\mu _t$	Target $\sigma ^2_t$	Benchmark
DGPs scenario I: models (24) and (25)				
$T=150$	0.0620	0.0011	0.0340	0.0021
$T=250$	0.1368	− 0.0021	0.0953	− 0.0002
$T=500$	0.3513	0.0008	0.3000	− 0.0004
$T=1000$	0.6473	− 0.0005	0.5914	− 0.0013
$T=2000$	0.8920	0.0079	0.8601	0.0015
DGPs scenario II: models (24) and (26)				
$T=150$	0.0231	− 0.0003	0.0151	0.0012
$T=250$	0.0039	0.0386	− 0.0005	− 0.0413
$T=500$	0.0406	− 0.0057	− 0.0088	0.0153
$T=1000$	0.3365	0.1925	0.2763	0.0362
$T=2000$	0.8941	0.3474	0.8292	0.0231
DGPs scenario III: models (24) and (27)				
$T=150$	0.0105	0.0013	0.0075	− 0.0001
$T=250$	0.0280	− 0.0008	0.0198	0.0039
$T=500$	0.0859	0.0075	0.0747	0.0004
$T=1000$	0.2385	0.0222	0.2084	− 0.0012
$T=2000$	0.4781	0.0305	0.4547	− 0.0007
DGPs scenario IV: models (25) and (26)				
$T=150$	0.0869	− 0.0001	0.0519	0.0086
$T=250$	0.1991	0.0102	0.1240	0.0009
$T=500$	0.4148	0.0110	0.3554	0.0017
$T=1000$	0.5509	0.0212	0.5008	0.0007
$T=2000$	0.9500	0.1172	0.9358	− 0.0004
DGPs scenario V: models (25) and (27)				
$T=150$	0.0241	0.0016	0.0102	0.0003
$T=250$	0.0555	0.0016	0.0254	0.0019
$T=500$	0.1242	0.0203	0.0791	− 0.0003
$T=1000$	0.1304	0.0188	0.0846	− 0.0005
$T=2000$	0.4522	0.1061	0.3717	0.0003
DGPs scenario VI: models (26) and (27)				
$T=150$	0.0038	− 0.0014	0.0005	− 0.0088
$T=250$	0.0503	− 0.0026	0.0360	− 0.0008
$T=500$	0.1557	− 0.0037	0.1327	− 0.0006
$T=1000$	0.3929	0.0037	0.3569	− 0.0004
$T=2000$	0.6860	0.0007	0.6579	− 0.0027

**Table 9:** Clustering results: average Adjusted Rand Index $(N=30; \, K=2)$

Clustering approach	Proposal	Target $\mu _t$	Target $\sigma ^2_t$	ACF-based
DGPs scenario I: models (24) and (25)				
$T=150$	0.1043	0.0054	0.0120	0.0113
$T=250$	0.1782	− 0.0092	0.0318	0.0024
$T=500$	0.4623	− 0.0081	0.0523	0.0031
$T=1000$	0.8185	− 0.0043	0.0905	0.0049
$T=2000$	0.9748	− 0.0012	0.2041	0.0025
DGPs scenario II: models (24) and (26)				
$T=150$	0.0244	0.0028	0.0164	0.0022
$T=250$	0.0547	− 0.0018	0.0538	− 0.0029
$T=500$	0.1650	0.0055	0.0736	− 0.0013
$T=1000$	0.5626	− 0.0026	0.1161	0.0051
$T=2000$	0.9030	− 0.0015	0.2340	0.0137
DGPs scenario III: models (24) and (27)				
$T=150$	0.0119	0.0017	0.0076	0.0012
$T=250$	0.0272	0.0039	0.0141	0.0044
$T=500$	0.1190	0.0018	0.0727	0.0053
$T=1000$	0.3091	− 0.0057	0.2192	0.0067
$T=2000$	0.6318	0.0068	0.5015	− 0.0029
DGPs scenario IV: models (25) and (26)				
$T=150$	0.1097	0.0065	0.1116	0.0025
$T=250$	0.2231	− 0.0016	0.2827	0.0101
$T=500$	0.5996	0.0050	0.4034	− 0.0033
$T=1000$	0.8955	0.0026	0.6007	0.0048
$T=2000$	0.9933	0.0031	0.8047	0.0009
DGPs scenario V: models (25) and (27)				
$T=150$	0.0377	0.0007	0.0164	− 0.0063
$T=250$	0.0570	0.0078	0.0288	− 0.0015
$T=500$	0.1321	− 0.0009	0.0846	− 0.0005
$T=1000$	0.3366	− 0.0012	0.2530	0.0020
$T=2000$	0.6259	− 0.0010	0.5317	− 0.0016
DGPs scenario VI: models (26) and (27)				
$T=150$	0.0220	− 0.0099	0.0059	− 0.0058
$T=250$	0.0760	0.0010	0.0475	0.0017
$T=500$	0.1911	− 0.0055	0.1378	0.0055
$T=1000$	0.3408	0.0071	0.2686	0.0040
$T=2000$	0.6299	− 0.0001	0.5704	0.0022

**Table 10:** Clustering results: average Adjusted Rand Index $(N=60; \,K=2)$

Clustering approach	Proposal	Target $\mu _t$	Target $\sigma ^2_t$	ACF-based
DGPs scenario I: models (24) and (25)				
$T=150$	0.0716	− 0.0013	0.0165	0.0037
$T=250$	0.2077	− 0.0030	0.0248	0.0038
$T=500$	0.5100	0.0030	0.0532	0.0087
$T=1000$	0.8544	− 0.0007	0.1030	0.0010
$T=2000$	0.9808	0.0041	0.1851	− 0.0024
DGPs scenario II: models (24) and (26)				
$T=150$	0.0219	0.0022	0.0153	− 0.0011
$T=250$	0.0599	0.0019	0.0387	0.0012
$T=500$	0.2238	0.0024	0.0637	0.0007
$T=1000$	0.5889	0.0032	0.1355	0.0002
$T=2000$	0.8988	0.0002	0.2322	0.0028
DGPs scenario III: models (24) and (27)				
$T=150$	0.0122	− 0.0011	0.0019	0.0024
$T=250$	0.0277	0.0021	0.0225	− 0.0024
$T=500$	0.1123	− 0.0019	0.0565	0.0009
$T=1000$	0.3552	0.0009	0.1881	0.0055
$T=2000$	0.6581	− 0.0006	0.4946	0.0011
DGPs scenario IV: models (25) and (26)				
$T=150$	0.1106	− 0.0023	0.0778	0.0047
$T=250$	0.2809	0.0026	0.1988	0.0044
$T=500$	0.6136	− 0.0003	0.4090	0.0010
$T=1000$	0.9259	− 0.0011	0.5869	0.0039
$T=2000$	0.9947	0.0060	0.8224	0.0012
DGPs scenario V: models (25) and (27)				
$T=150$	0.0286	− 0.0001	0.0125	− 0.0024
$T=250$	0.0610	0.0001	0.0529	0.0064
$T=500$	0.1661	0.0015	0.1297	0.0044
$T=1000$	0.3835	0.0050	0.3066	0.0022
$T=2000$	0.6447	0.0063	0.5687	0.0013
DGPs scenario VI: models (26) and (27)				
$T=150$	0.0010	− 0.0022	0.0044	0.0030
$T=250$	0.0031	0.0003	0.0049	− 0.0013
$T=500$	0.0170	0.0012	0.0066	− 0.0002
$T=1000$	0.0262	− 0.0021	0.0184	0.0009
$T=2000$	0.1192	0.0005	0.0723	− 0.0005

## Appendix 2: The GAS model

The GAS model is based on the assumption that, for each *n*-th unit, the time series variable $y_{n,t}$ is generated by the following observation density $p(\cdot )$:

28 $$\begin{aligned} y_{n,t} \sim p(y_{n,t} \vert f_{n,j,t}, {\mathcal {F}}_{n,t}; \theta _n) \end{aligned}$$

where $\theta _n$ is a vector of static parameters, ${\mathcal {F}}_{n,t}$ is the information set at time *t*, $f_{n,j,t}$ are the *J* $(j=1,\ldots ,J)$ time–varying parameters depending on the probability distribution. The model’s information set at time *t*, called ${\mathcal {F}}_{n,t}$, is obtained by the previous realizations of the time series $y_{n,t}$ and its time-varying parameters $f_{n,j,t}$. The Generalized Autoregressive Score of order one, the *GAS*(1, 1), can be written as:

29 $$\begin{aligned} f_{n,j,t} = \omega _{n,j} + {\mathbf {A}}_{n, j} s_{n, j, t-1} + {\mathbf {B}}_{n, j} f_{n, j,t-1} \end{aligned}$$

where $\omega _{n,j}$ is a real vector and ${\mathbf {A}}_{n,j}$ and ${\mathbf {B}}_{n,j}$ are diagonal matrices. All the scalar parameters $\omega _{n,j}, {\mathbf {A}}_{n,j}, {\mathbf {B}}_{n,j}$ are collected in the vector $\theta _n$. Moreover, $s_{n,j,t}$ is the *scaled* score of the conditional density (28) in a time *t* in relation to a *j*-th parameter of the *n*-th time series:

30 $$\begin{aligned} s_{n,j,t} = S_{n,t}\cdot \nabla _{n,j,t} \end{aligned}$$

with $\nabla _{n,j,t}$ being the conditional score:

31 $$\begin{aligned} \nabla _{n,j,t} = \frac{\partial \log p(y_{n,t} \vert f_{n,j,t}, {\mathcal {F}}_{n,t}; \theta )}{\partial f_{n,j,t}} \end{aligned}$$

and $S_{n,t}$ a scaling matrix that depends on the probability distribution, since it is usually set equal to the Fisher information matrix or to the Identity matrix in the case of no scaling.

In other words, in the GAS model we suppose that the evolution of the time-varying parameter vector $f_{n,j,t}$ depends both on a vector $s_{n,j,t}$, proportional to the score of the density, and on autoregressive component.

A useful feature of the GAS model is that the vector $\theta _n$ is obtained by a maximum likelihood estimator (Creal et al. 2013).

## Appendix 3: Compromise computation with DISTATIS

The Distance STATIS (DISTATIS, see Abdi et al. 2005) approach is used with the aim of synthesizing many distance matrices computed on the same set of statistical units. The main idea behind DISTATIS is to transform each of these distance matrices into a cross-product matrix and, then, synthesise the several obtained matrices with a STATIS algorithm (Escoufier 1980; Thiébaut et al. 1977). Therefore, the final result of DISTATIS approach is the definition of a so-called compromise matrix as the best synthesis of the original distance matrices.

Thus, starting from $K(k=1,\ldots ,K)$ distance matrices ${\mathbf {D}}_k$, the first step of DISTATIS is to transform each distance matrix ${\mathbf {D}}_k$ into a cross-product matrix $\varvec{\tilde{S}}_{k}$:

32 $$\begin{aligned} \varvec{\tilde{S}}_{k}=-\frac{1}{2} \Xi {\mathbf {D}}_{k} \varvec{\Xi } \end{aligned}$$

where $\Xi =I_N-1_N \mathbf {m}^{\prime }$, $I_N$ is the identity matrix of dimension *N* (where *N* is the number of the observed statistical units), $1_N$ is a vector of ones and $\mathbf {m}$ is a vector of *N* equal elements $m_n=1/N\text { }(\text {for } n=1,\ldots ,N)$.

The initial transformation (32) is necessary because the original distance matrices cannot be directly analyzed with STATIS since they are not positive semi-definite. This transformation is particularly relevant when we start from Euclidean distance matrices because, in this case, it is completely reversible, i.e. each Euclidean distance matrix can be perfectly reconstituted from its corresponding cross-product matrix and vice-versa (Abdi et al. 2012). The matrices $\varvec{\tilde{S}}_{1}, \ldots , \varvec{\tilde{S}}_{K}$ are often normalized prior to the analysis such that, for example, the sum of their squared elements is equal to one or that they have a first eigenvalue equal to one.

In the second step, we search a compromise matrix. The compromise matrix is a cross-product matrix that gives the best compromise of the cross-product matrices. It is obtained as a weighted average of these matrices. Therefore, it is necessary to derive an optimal set of weights, by considering the degree of similarity among the *K* cross-product matrices $\varvec{\tilde{S}}_{1}, \ldots , \varvec{\tilde{S}}_{K}$.

The degree of similarity between two generic cross-product matrices $\varvec{\tilde{S}}_{k}$ and $\varvec{\tilde{S}}_{k\prime }$ is computed by means of the $R_V$ coefficient, defined as:

33 $$\begin{aligned} R_{V}(k, k^{\prime })=\left[ c_{k, k^{\prime }}\right] =\frac{ \text{ trace } \left( \varvec{\tilde{S}}_{k}^{\prime } \varvec{\tilde{S}}_{k^{\prime }}\right) }{\sqrt{{\text {trace}}\left( \varvec{\tilde{S}}_{k}^{\prime } \varvec{\tilde{S}}_{k}\right) {\text {trace}}\left( \varvec{\tilde{S}}_{k^{\prime }}^{\prime } \varvec{\tilde{S}}_{k^{\prime }}\right) }} \end{aligned}$$

$R_V(k, k^{\prime })$ coefficients for each couple *k* and $k^{\prime }$ are the generic elements of a so-called *cosine* matrix $\mathbf {C}$. By construction the $R_V$ coefficients fall in the interval $[-1, 1]$. This means that, considering two distances matrices *k* and $k^{\prime }$, we have that they perfectly agree on the position of the units if $c_{k, k^{\prime }}=1$, provide opposite results in the case $c_{k, k^{\prime }}=-1$ and are orthogonal if $c_{k, k^{\prime }}=0$.

To find the optimal weights to use for calculating the compromise matrix, DISTATIS computes the eigen-decomposition of the cosine matrix $\mathbf {C}$:

34 $$\begin{aligned} \mathbf {C}=\mathbf {P} \Lambda{ \mathbf {P}}^{\prime }\, {\text { given }} \,{\mathbf {P}}^{\prime }{ \mathbf {P}}={\mathbf {I}} \end{aligned}$$

where $\mathbf {P}$ is the matrix of eigenvectors $\mathbf {p}_1, \ldots ,{ \mathbf {p}}_K$ and $\Lambda$ is the diagonal matrix of the eigenvalues of $\mathbf {C}$. Let us define the optimal weights vector $\varvec{\alpha }$, with generic element $\alpha _k$ $(k=1,\ldots ,K)$, computed as:

35 $$\begin{aligned} \varvec{\alpha }=\left( \mathbf {1}^{\prime }{ \mathbf {p}}_{1}\right) ^{-1} {\mathbf {p}}_{1}, \end{aligned}$$

where $\mathbf {p}_{1}$ is the first eigenvector of $\mathbf {C}$. Therefore, the compromise is computed as:

36 $$\begin{aligned} \varvec{\tilde{S}} = \sum _{k=1}^{K} \alpha _k \varvec{\tilde{S}}_k \end{aligned}$$

Starting from the cross-product matrix $\varvec{\tilde{S}}$ it is possible to obtain the following Euclidean squared distance matrix $\varvec{\tilde{D}}$ (see Salkind 2006):

37 $$\begin{aligned} \varvec{\tilde{D}} = \varvec{\tilde{s}} 1_N^{\prime } + 1_N \varvec{\tilde{s}}^{\prime } - 2 \varvec{\tilde{S}} \end{aligned}$$

with $\varvec{\tilde{s}}$ being a vector containing the diagonal element of $\varvec{\tilde{S}}$, i.e. $\varvec{\tilde{s}}={\text {diag}}\left( \varvec{\tilde{S}}\right)$. Therefore, the (37) represents the consensus matrix between two or more distance matrices.

## Footnotes

1 Note that, following (5) it should be ${\mathbf {D}}_{n,1}$. However, to not abuse with notation, we write ${\mathbf {D}}_{n,1} ={\mathbf {D}}_n$.

2 In the case in which $J\ge 3$, instead, we have that by concatenating the ${\text{vec}}\left( \mathbf {L}_n\right)$ we obtain a matrix $\mathbf {X}$ of dimension $[J(J-1)]/2\times N$, thus turning into a 2D clustering problem.

3 The boxplots of all the considered time series lengths are available upon request.

4 Also in this case, the entire set of the boxplots of all the considered time series lengths is available upon request.

5 The considered cities are the following: Ahmedabad, Amaravati, Bengaluru, Chandigarh Coimbatore, Delhi, Gurugram, Guwahati, Hyderabad, Jaipur, Kolkata, Mumbai, Patna, Thiruvananthapuram and Visakhapatnam. Other cities have been removed because of missing values

6 Data about monitoring station can be retrieved at the following link https://cpcb.nic.in/. The final dataset at city level is available on request.
